# Acceptability of Interventions Delivered Online and Through Mobile Phones for People Who Experience Severe Mental Health Problems: A Systematic Review

**DOI:** 10.2196/jmir.5250

**Published:** 2016-05-31

**Authors:** Natalie Berry, Fiona Lobban, Richard Emsley, Sandra Bucci

**Affiliations:** ^1^ Health eResearch Centre (HeRC) Institute of Population Health University of Manchester Manchester United Kingdom; ^2^ School of Psychological Sciences Faculty of Medical and Human Sciences University of Manchester Manchester United Kingdom; ^3^ Spectrum Centre for Mental Health School of Health and Medicine Lancaster University Lancaster United Kingdom; ^4^ Centre for Biostatistics Institute of Population Health University of Manchester Manchester United Kingdom

**Keywords:** mHealth, eHealth, severe mental health, psychosis, bipolar disorder, personality disorder, severe mental health problems (SMI), acceptability

## Abstract

**Background:**

Psychological interventions are recommended for people with severe mental health problems (SMI). However, barriers exist in the provision of these services and access is limited. Therefore, researchers are beginning to develop and deliver interventions online and via mobile phones. Previous research has indicated that interventions delivered in this format are acceptable for people with SMI. However, a comprehensive systematic review is needed to investigate the acceptability of online and mobile phone-delivered interventions for SMI in depth.

**Objective:**

This systematic review aimed to 1) identify the hypothetical acceptability (acceptability prior to or without the delivery of an intervention) and actual acceptability (acceptability where an intervention was delivered) of online and mobile phone-delivered interventions for SMI, 2) investigate the impact of factors such as demographic and clinical characteristics on acceptability, and 3) identify common participant views in qualitative studies that pinpoint factors influencing acceptability.

**Methods:**

We conducted a systematic search of the databases PubMed, Embase, PsycINFO, CINAHL, and Web of Science in April 2015, which yielded a total of 8017 search results, with 49 studies meeting the full inclusion criteria. Studies were included if they measured acceptability through participant views, module completion rates, or intervention use. Studies delivering interventions were included if the delivery method was online or via mobile phones.

**Results:**

The hypothetical acceptability of online and mobile phone-delivered interventions for SMI was relatively low, while actual acceptability tended to be high. Hypothetical acceptability was higher for interventions delivered via text messages than by emails. The majority of studies that assessed the impact of demographic characteristics on acceptability reported no significant relationships between the two. Additionally, actual acceptability was higher when participants were provided remote online support. Common qualitative factors relating to acceptability were safety and privacy concerns, the importance of an engaging and appealing delivery format, the inclusion of peer support, computer and mobile phone literacy, technical issues, and concerns about the impact of psychological state on intervention use.

**Conclusions:**

This systematic review provides an in-depth focus on the acceptability of online and mobile phone-delivered interventions for SMI and identified the need for further research in this area. Based on the results from this review, we recommend that researchers measure both hypothetical and actual acceptability to identify whether initial perceptions of online and mobile phone-delivered interventions change after access. In addition, more focus is needed on the potential impact of demographic and clinical characteristics on acceptability. The review also identified issues with module completion rates and intervention use as measures of acceptability. We therefore advise researchers to obtain qualitative reports of acceptability throughout each phase of intervention development and testing. Further implications and opportunities for future research are discussed.

## Introduction

The exact definition of severe mental health problems (SMI) is inconsistent in the literature. Discussions have proposed that the term should be applied to describe the duration and levels of functioning in an individual, rather than focusing on specific diagnoses [1,2]. However, much of the psychological research in SMI specifically uses diagnostic criteria for participant recruitment [3]. Due to the reliance on diagnosis for recruitment in this field of research, we used a diagnostic definition of SMI for this review. Online and mobile phone-delivered interventions have the potential to improve access to evidence-based interventions for SMI; therefore, we used the Improving Access to Psychological Therapies (IAPT) initiative definition of SMI. IAPT is a United Kingdom-based National Health Service (NHS) program, which aims to increase access and availability of evidence-based psychological therapies and includes diagnoses of psychosis, bipolar disorder, and personality disorders as SMI [4]. The initiative is in line with current recommendations that people experiencing SMI be provided access to evidence-based psychological interventions, in addition to prescribed medications [5-7].

A range of barriers, including perceived stigma [8], uncertainty among practitioners about clinical effectiveness [9], cost pressures, and lack of trained facilitators [10], means that many people who could benefit from psychological interventions are often unable or unwilling to access them. Limited availability and access to psychological therapies for SMI was reflected in a survey by the charity Mind, which reported that 20% of respondents had waited for >1 year to access psychological therapies and <30% had received access within 3 months of referral [11]. In addition, a recent report by the NHS’s Mental Health Taskforce highlighted that respondents’ “most important” priorities for NHS mental health service improvement were early support and intervention for people experiencing SMI and increased access to psychological therapies [12]. In an attempt to reduce barriers and provide increased access to helpful interventions, researchers have investigated the role that novel technologies could play in the provision of evidence-based interventions for people with SMI.

Interventions delivered online (ie, websites) and via mobile phones (ie, smartphones, text messages, alerts, and apps) have been reported to be acceptable and show potential efficacy for the enhancement of self-care practices in individuals across a broad field, including diabetes [13,14], cancer [15], coronary heart disease [16], and psoriasis [17]. Additionally, online and mobile phone-delivered approaches have been used to implement evidence-based interventions for the promotion of health-related behaviors such as smoking cessation [18,19], physical activity [20], and weight reduction [21]. More recently, interventions have been delivered online and via mobile phones in an attempt to, among other things, reduce barriers associated with accessing mental health care [22] and to empower service users with greater choice and control over their health care needs [23]. Online and mobile phone-delivered interventions have been shown to be feasible, acceptable, and effective for depression and anxiety disorders [24,25], eating disorders [26], and substance use [27]; these interventions have since been extended to people who experience psychosis [28,29], bipolar disorder [30], and personality disorders [31], or the so-called severe mental health problems (SMI).

Levels of Internet use among people with mental health problems, including SMI, are similar to that of the general population [32]. In a survey of service users in a community psychiatric program, 59.3% of respondents reported using the Internet and 85.7% reported using mobile phones [33]. Additionally, a survey-based study for individuals with SMI reported that 72% of participants surveyed owned a mobile phone, while some expressed an interest in receiving health care services, such as appointment and medication reminders, through mobile devices [34]. A meta-analysis conducted in 2015 reported mobile phone ownership of around 81.4% in people with psychosis, reflecting similar ownership to that in the general population [35]. This meta-analysis also reported that many of the people who were surveyed expressed favorable attitudes toward mobile phone-delivered self-management strategies, for example, symptom monitoring, appointment and medication reminders, and providing an avenue for service user-provider communication. However, a more recent study of 100 individuals experiencing SMI reported high levels of traditional mobile phone ownership (85%) but lower levels of smartphone ownership (37%) [36]. The comparatively lower rates of smartphone ownership suggest that some individuals with SMI might be excluded from being able to receive interventions via smartphones; however, current levels of interest and traditional mobile phone usage in this population suggest that many would have the capabilities needed to receive online and mobile phone-delivered interventions.

Previous systematic reviews of online and mobile phone-delivered interventions for people with SMI have tended to have broad focus on acceptability, feasibility, and efficacy, rather than provide an in-depth review of one outcome. For example, a review from 2014 examined the effectiveness of online, social media, and mobile technologies for people with psychosis [28]. The authors found that interventions delivered online and via mobile devices are often feasible and acceptable, and show potential efficacy among this group. Another review of e-mental health self-management for psychotic disorders found that individuals were willing to engage with online interventions and that such approaches can be effective for the promotion of self-management strategies [29]. More recently, a review of mobile device and eHealth interventions for people who have received a psychotic or bipolar-related diagnosis reported that interventions delivered via these modalities are both feasible and acceptable [37]. Finally, a 2015 review examined the feasibility of smartphone apps for individuals with schizophrenia [38]. The authors highlighted that the number of studies using smartphone apps for schizophrenia are limited, but they concluded that there was evidence for high feasibility due to satisfaction reports and levels of engagement. The lack of high-quality, large-scale, definitive research prevents any conclusive statements from being made. The conclusion that online and mobile phone-delivered interventions are acceptable for people with SMI has been largely based on module completion rates, intervention use, and participants’ views. However, reviews have not captured the complex nature of acceptability. Specifically, reviews have not included studies investigating participant attitudes, views, and interest in interventions delivered online and via mobile phones. Additionally, potential factors that may influence acceptability, such as demographic, clinical, and intervention characteristics, have yet to be synthesized. Finally, common qualitative themes relating to the acceptability of online and mobile phone-delivered interventions for SMI have not yet been identified in systematic reviews.

In order to more closely examine acceptability, we sought to examine both the hypothetical and actual acceptability of interventions delivered online and via mobile phones for people with SMI. We define hypothetical acceptability as the acceptability of online and mobile phone-delivered interventions prior to or without an intervention being delivered, measured by participants’ interest in and willingness to engage with these interventions. We define actual acceptability as the acceptability of an intervention that participants have received online or via mobile phones, which can be measured by module completion rates, intervention use, and participant views after an intervention has been delivered.

Therefore, this systematic review aimed to 1) explore whether interventions delivered online and via mobile phones are hypothetically or actually acceptable for people with SMI, 2) investigate whether participant and intervention-related factors influence acceptability, and 3) identify common participant views about acceptability from qualitative studies.

## Methods

### Search Strategy

This review was conducted in accordance with the Preferred Reporting Items for Systematic Reviews and Meta-Analyses (PRISMA) [39]. We identified studies for inclusion through searching the electronic databases PubMed, Embase, PsycINFO, CINAHL, and Web of Science. The first set of search terms were related to SMI: “psychosis” OR “psychotic” OR “psychoses” OR “schizophr*” OR “schizoaffective” OR “bipolar disorder” OR “mood disorder” OR “personality disorder” OR “severe mental illness” OR “serious mental illness” OR “severe mental health” OR “serious mental health” OR “SMI”. The second set of search terms were related to online and mobile technologies: “computer” OR “technolog*”, OR “digital” OR “internet” OR “online” OR “website” OR “web-based” OR “mobile” OR “phone” OR “smartphone” OR “text message” OR “SMS” OR “mHealth” OR “eHealth”. These sets of search terms were linked with the Boolean operator AND.

To increase the likelihood of obtaining all of the relevant studies in the area, we took the following steps: 1) we included unpublished materials such as conference abstracts in the search; where abstracts were relevant, we contacted the lead authors for full results, 2) we used the “cited-by” function in Google Scholar to identify any eligible papers that had cited the included studies, 3) we screened references lists of included studies to gather any papers that the search terms had not identified, and 4) we contacted key authors in the area who were identified as potentially having further unpublished results.

**Figure 1:**
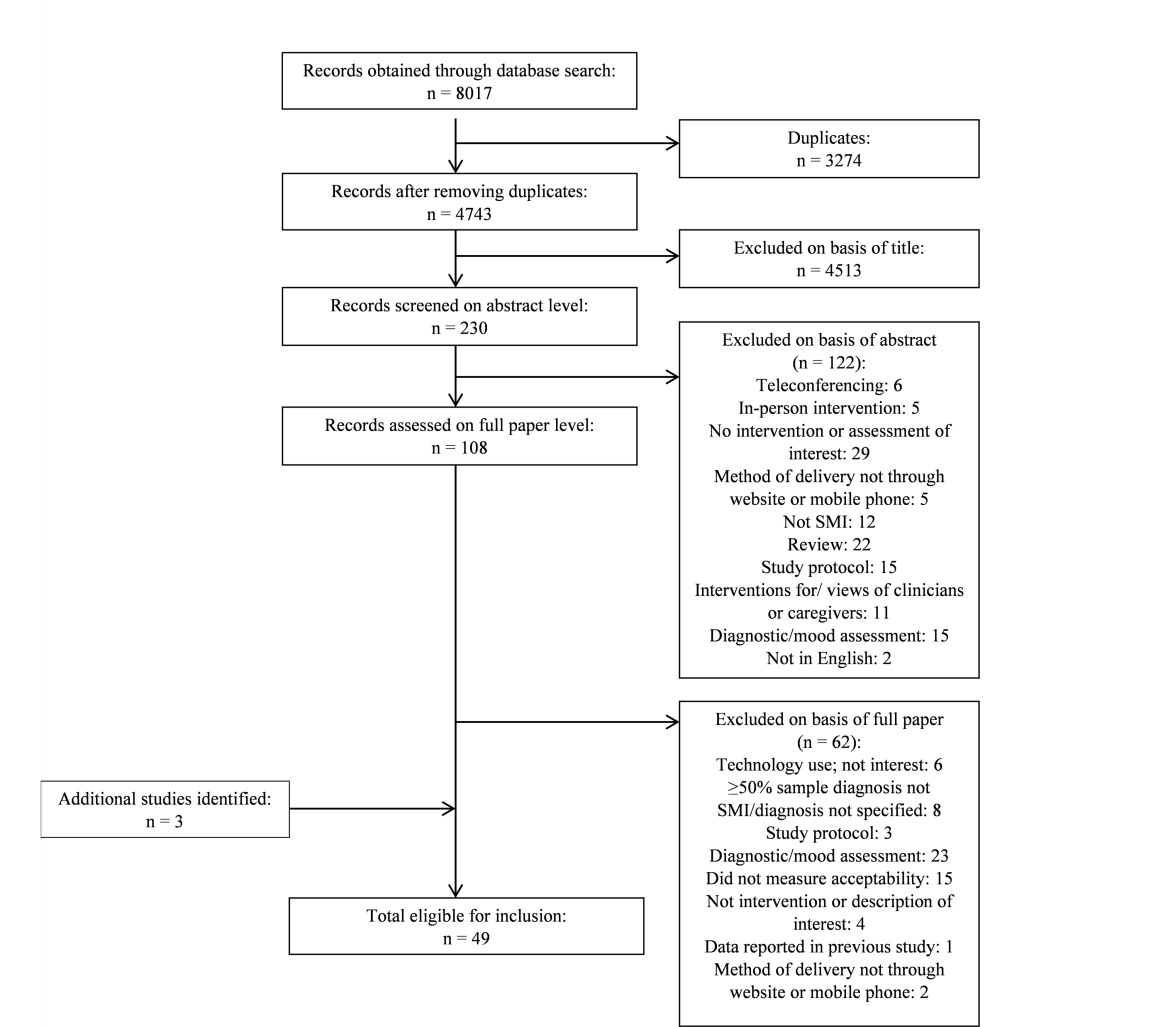
Flow diagram of systematic search for interventions delivered online and through mobile phones for people with severe mental health problems (SMI).

We produced a flowchart of each stage of the database search, with the search yielding a total of 8017 papers (Figure 1).

### Eligibility Criteria

We conducted full database searches in April 2015, with the inclusion and exclusion criteria identified prior to the collection period. Due to the relatively new nature of the field of the Internet and mobile phones and mental health, we considered only studies from 2005–2015. We included studies if they recruited participants with a diagnosis of psychosis, bipolar disorder, or personality disorder. Studies where these diagnoses were self-reported (not confirmed by a clinician or through an initial assessment) were still included due to Web-based recruitment strategies often used in the field. We defined interventions as a method used online or via a mobile phone with the aim of modifying a participant’s behavior or psychological well-being. Therefore, we aimed to provide a broad range of interventions, from more simplistic interventions, such as medication and appointment reminders, to more complex interventions, such as cognitive behavioral therapy and interactive psychoeducation. We included studies if they involved the delivery of an intervention online or via a mobile phone or if they investigated participants’ interest in and willingness to receive interventions delivered in these formats. Supported interventions (where participants were supported by a trained facilitator or where online and mobile phone-delivered interventions were implemented in conjunction with face-to-face therapy) were also included because some of the studies offered optional support and often continued face-to-face treatment as usual. Finally, we included studies if they measured acceptability in the form of module completion rates, intervention use, or participant views. Therefore, we included qualitative, quantitative, or mixed-methods study designs.

We excluded studies if the intervention was solely focused on diagnostic assessments, mood assessments, or symptom monitoring where feedback was not provided to participants. In addition, we excluded studies involving telepsychiatry, for example, video and telephone calls. We included studies that involved a combination of caregivers and service users but we report characteristics and outcomes only for the participants from the service user sample. Studies that involved mixed samples were excluded if ≤50% of the participants sampled had a diagnosis of psychosis, bipolar disorder, or personality disorder. For those where >50% of participants had 1 of these diagnoses, we contacted the authors of these papers for separate results for participants with a diagnosis of psychosis, bipolar disorder, and personality disorders.

A key aim of this systematic review was to determine factors (eg, demographic and clinical characteristics) that could influence the acceptability of online and mobile phone-delivered interventions for SMI. Therefore, we screened included studies for information about the analysis and findings relating to potential predicators of acceptability. We considered relationships between factors and acceptability as significant if they were below the .05 level of significance. However, due to the early nature of this research, we also report associations on the 10% level.

### Study Selection

We excluded studies on the title level if there was no mention of online or mobile phone-delivered interventions or mental health. The first author and an independent researcher screened the 230 paper abstracts for eligibility, with a moderate level of agreement obtained (κ=.66). This moderate level of agreement was primarily due to the first author being overly inclusive while screening abstracts due to the secondary outcome nature of acceptability. The research team resolved any disagreements until a consensus was reached about study inclusion. The first author screened full texts before the research team discussed and agreed on final papers for inclusion. We identified 3 additional studies through contacting authors and reference screening. We contacted 4 authors for clarification because their studies included participants with a diagnosis of major depressive disorder, and 3 of these authors provided additional unpublished material, which we have included in this review. The final number of included studies after screening on title, abstract, and full paper level was 49.

## Results

We explored the hypothetical acceptability of online and mobile phone-delivered interventions for SMI in 7 studies [34,40-46] (1821 participants; sample size range 51–1237). The mean age of participants in studies where mean age was reported [34,40,41,44,45] (n=4) was 32.73 years (range 18.33–46). Studies were conducted in the United States [34,40,41,45] (n=3), the United Kingdom [42,46] (n=2), Canada [44] (n=1), and India [43] (n=1). Multimedia Appendix 1 details the study and participant characteristics.

The actual acceptability of online and mobile phone-delivered interventions for people with SMI was measured in 42 studies [31,40,47-86] (2226 participants; sample size range 4–311). The mean age of participants across the studies where mean age was reported [31,40,47-51,53,54,57,59, 60,62-68,70-74,77-80,82-86] (n=27) was 40.61 years (range 27–48.8). The average number of intervention modules per sessions was 8.5 (range 4–20), while the average intervention duration was 17 weeks (range 2–78). The majority of the studies were conducted in the United States [31,40,60,62-64,66,68, 72,73,78-81,86] (n=15) and Australia [47-49,59,70,71,74-76] (n=9), while the remainder were from Finland [50-55] (n=6), the United Kingdom [56-58,82,83] (n=5), the Netherlands [61,77,84,85] (n=4), Canada [65] (n=1), Sweden [67] (n=1), and the Czech Republic [69] (n=1). Multimedia Appendix 2 details the study and participant characteristics.

### Hypothetical Acceptability

Multimedia Appendix 3 presents results for the hypothetical acceptability of online and mobile phone-delivered interventions for SMI. The results reported focused on 1) the proportion of participants who agreed that they would be interested in receiving online and mobile phone-delivered interventions, 2) the impact of demographic characteristics on hypothetical acceptability, 3) whether participant levels of interest in online and mobile phone-delivered interventions differed between email and text message delivery, and 4) geographical differences in hypothetical acceptability.

The hypothetical acceptability of obtaining support via social media websites was relatively high (75.5%), and two-thirds of participants (64%) were willing to have clinicians contact them via social media during symptom emergence [41] (A Rizvi, MA, written communication, May 2015). However, levels of interest in receiving information and support varied between social media platforms (85% YouTube; 58% Facebook; 39% Twitter) [44]. Interest in mobile phone check-ins to inform health care providers about symptoms was relatively low (14.4% to 41%) [34,40,43] (D Ben-Zeev, PhD, written communication, May 2015), while interest in receiving text messages from health care providers was moderate to high (45% to 76%) [45,46]. Additionally, the hypothetical acceptability of online and mobile phone-delivered appointment and medication reminders varied extensively between studies (26% to 92.5%) [34,40,42-45] (personal communication with D Ben-Zeev, May 2015).

Participant interest in mobile phone-delivered information about treatment, services, and psychoeducation was relatively low (31% to 48.1%) [34,40,43] (personal communication with D Ben-Zeev, May 2015). However, a 2015 study specifically recruiting young people with first episode psychosis reported that 90% of participants liked the idea of receiving information about physical and mental health online or via mobile phones [44].

#### Impact of Demographic Characteristics on Hypothetical Acceptability

No significant relationships were reported between interest in text message and email-delivered interventions and age, sex, race, and employment status [42,45]. However, in 1 study, there was an association at the 10% level between age and interest in receiving medication reminders via text messages (*P*=.06) [42]. The mean and median age of participants was <35 years in 2 studies [41,44] (personal communication with A Rizvi, May 2015) and ≥35 years in 3 studies [34,40,43,45]. Interest in online and mobile phone-delivered appointment and medication reminders was higher in studies where the average age of participants was <35 years (56.7% to 92.5%) in comparison with >35 years (26% to 58%).

#### Hypothetical Acceptability of Interventions Delivered via Text Messages Versus Emails

In 2 studies, participants were asked whether they would prefer to receive appointment and medication reminders, check-ins with providers, and health-related information via telephone calls, text messages, or emails. In both studies, participants stated a preference for receiving telephone calls (72.6% to 81%) in comparison with text messages (8.5% to 36%) and emails (1.5% to 21%) [34,40,43] (personal communication with D Ben-Zeev, May 2015). In 2 other studies, participants also reported a preference for medication and appointment reminders delivered via text messages (40% to 92.5%) in comparison with email (26% to 79.1%) [44,45].

#### Differences in Hypothetical Acceptability Geographically

A questionnaire developed for participants in the United States was adapted for use in India [34,43]. A larger proportion of participants in the United States were interested in using the Internet and mobile phones for check-ins with providers (39.5%) and appointment and medication reminders (41.5%) compared with participants in India (check-ins: 14%; medication and appointment reminders: 27%) [34,40,43] (personal communication with D Ben-Zeev, May 2015). However, interest in mobile phone-delivered health information and psychoeducation was of more interest to participants in India (44%) than to those in the United States (31%).

### Actual Acceptability

Multimedia Appendix 4 presents results for the actual acceptability of online and mobile phone-delivered interventions for SMI. The results reported focused on 1) intervention satisfaction ratings reported by participants, 2) intervention use and module completion rates, 3) the impact of demographic and clinical characteristics on actual acceptability, and 4) a comparison of the actual acceptability between supported and unsupported online and mobile phone-delivered interventions.

Some studies measured the actual acceptability of online and mobile phone-delivered interventions for SMI through satisfaction ratings. The proportion of participants who were satisfied with the ease of use, perceived helpfulness, and perceived usefulness of the interventions, and were willing to recommend the intervention to others, was moderate to high (41% to 90.6%), although the majority of studies tended to report values of around 75% [40,47,52,60,66-68,77-79,85]. Other studies measured actual acceptability through satisfaction ratings on Likert scales. Ratings for overall satisfaction and perceived helpfulness and usefulness were on the upper ends of the Likert scales, while moderate to high ratings were reported for ease of use [32,50,62-65,80,84,86] (personal communication with L Warner, June 2015).

Intervention use was also included as a measure of actual acceptability. In the HORYZONS study, 60% of participants used the intervention for the full 4 weeks and 70% used it for at least 3 weeks, as measured by the number of intervention log-ins [47]. Additionally, the FOCUS system [40] was used by participants on 86.5% of the study days [60], while 71% of participants in the WEGWEIS project used every feature on the website [85]. In the SOAR study, every person who participated was reported to have engaged with the website (used the education material on at least 4 visits and contributed to the forum on at least 13 visits) [78,79]. However, rates of engagement with the MyRecoveryPlan website were relatively low after 3 weeks (program only: 9%; program plus coaching: 38%) [81]. Participant response rates to assessments and prompts ranged from 65% to 93.33% [32,62,63,72,73,86].

Actual acceptability was also measured through module completion rates and session attendance. Between 45% and 81% of participants completed at least half the modules of their assigned intervention [47,57,59,66,71,76], while 81% of participants in the Mental.Net project attended at least 3 of the 5 sessions [53]. Additionally, the average program completion rate of participants who remained in the LWB trial was 60% [83].

#### Impact of Demographic and Clinical Characteristics on Actual Acceptability

Only 3 studies investigated whether demographic characteristics influenced actual acceptability. Of these studies, 2 reported no significant relationships between actual acceptability and age, sex, or education level [54,63]. However, 1 study reported significantly higher workbook completion rates by participants who were female and older, although this was not found for educational attainment [75,76].

Several studies explored the relationship between participant psychological state and actual acceptability. The majority of these studies reported that the presence and severity of symptoms associated with SMI, for example, anxiety, depression, mania, and reduced cognitive functioning, did not predict participant satisfaction, module completion rates, and intervention use [54,60,63,75,76,86]. However, in 1 study, there was a positive association at the 10% level between completion rates and depressive symptoms (*P*=.06) and perceived helpfulness and baseline manic symptoms (*P*=.07) [78]. Additionally, there was a significant positive relationship between participant access and use of the SOAR website and severity of positive symptoms [79].

#### Actual Acceptability of Supported Versus Unsupported Internet-Delivered Interventions

Comparisons of actual acceptability between supported and unsupported Internet-delivered interventions for SMI were explored in 2 studies. In the MyRecoveryPlan and BEP studies, participants were assigned to receive access to either the basic programs without support or the programs plus remote coaching and support via email to help participants when using the intervention [75,76,81]. Participant completion rates in the BEP study and usage of the MyRecoveryPlan website were significantly higher in the remote support conditions than in the unsupported conditions (*P* <.05).

### Common Qualitative Themes

Some projects included qualitative studies to investigate participant views about the acceptability of online and mobile phone-delivered interventions for SMI and ideas for future developments to increase acceptability [49,51,53-56,58,61,62,69,70,74,75,82,84,85] (Multimedia Appendix 5). To aid conceptual understanding, we collected common participant quotes relating to acceptability by screening the papers and creating a set of key themes that emerged. The key common themes identified were 1) concerns about the safety, privacy, and security of online and mobile phone-delivered interventions, 2) the importance of an engaging and appealing intervention delivery format, 3) participants’ desire for the inclusion of remote peer support, 4) individual differences in computer and mobile phone literacy and technical issues as potential barriers to acceptability, and 5) the potential impact of psychological state on motivation to engage.

#### Participant Safety

Participant perceptions about the safety, confidentiality, and privacy of online and mobile phone-delivered interventions were noted in several studies [48,49,54,56,58,61,62]. Participants in the HORYZONS project felt the social networking component was safe and confidential due to its anonymous nature, restricted access, and expert moderation [48,49]. However, concerns about confidentiality were raised in Beating Bipolar focus groups, and 2 participants in the randomized controlled trial felt their privacy had been compromised through accessing the program on public computers [56,58]. Focus groups in the PCR project also revealed privacy and security concerns [61], while some participants using Mental.Net were worried about the confidentiality of the computerized delivery method [54]. Finally, the pilot trial for PRISM revealed that some participants were concerned about what they would say if other people asked what the device was for [62].

#### Engaging and Appealing

Comments relating to the appearance and layout of online and mobile phone-delivered interventions for SMI were evident in the included studies [40,49,51,55,56,70,74,82,84]. Many participants were satisfied with the clarity, layout, and appearance of the Mental.Net, Beating Bipolar, and WEGWEIS websites [55,56,84]. The inclusion of interactive components was generally popular. Participants in the HORYZONS project felt the interactive modules were fun; however, some were overwhelmed by the number of components [49]. Participants valued the interactive components and video and audio features of Beating Bipolar [56] and ORBIT [74], and recommended more interactive and video features on the Mental.Net and WEGWEIS websites [51,55,84]. The visual aids and pictures in FOCUS received positive comments [40], while participants liked the interactive mood monitor and flash objects on MoodSwings [70]. Finally, focus group discussions for LWB revealed that the idea of an online intervention was popular due its potential for interactive elements [82].

#### Peer Support

Online and mobile phone-delivered interventions for SMI sometimes include the opportunity for remote peer-to-peer communication. Participants in the HORYZONS project expressed that they liked the inclusion of social networking [48,49]; however, participants using Beating Bipolar were disappointed with the lack of discussion forum activity [58]. In the modification stage of the Mental.Net website, participants suggested the provision of a discussion forum [55], while participants in the LWB focus groups requested the option to communicate with peers online [82].

#### Computer and Mobile Phone Literacy and Technical Issues

Due to the technical nature of online and mobile phone-delivered interventions, some participants with low technology literacy may not find interventions delivered in this format acceptable. A small number of participants using Beating Bipolar reported low engagement due to poor computer literacy [58], while nurses in the Mental.Net project noted that disruptions were often related to insufficient technology skills [54].

Issues relating to technical functioning were also raised as potentially affecting acceptability. Nurses in the Mental.Net project felt some sessions were disrupted by technical problems such as network access [54], while participants in the modification stage reported technical issues such as inactive links [55]. Some components in the PCR study did not function adequately [61], while LWB focus group participants revealed a reluctance to engage with a website containing technical errors [82].

#### Impact of Psychological State on Engagement

Concerns were also raised about the influence of psychological state on intervention engagement. Some participants involved in the development of Beating Bipolar and LWB expressed concerns that people experiencing severe symptoms would not be able to engage with it [56,82]. In the Beating Bipolar testing phase, some participants felt their engagement was reduced by low mood, while others felt that low mood increased their engagement [58]. Participants who did not complete the BEP believed this was due to experiencing acute symptoms, which left them unable to engage [75].

## Discussion

The aim of this review was to explore both the hypothetical and actual acceptability of interventions delivered online and via mobile phones in SMI. The results support the assertion made in previous reviews that the actual acceptability of online and mobile phone-delivered interventions for SMI is relatively high [28,29,37,38]. However, unique to this review are the findings demonstrating the hypothetical acceptability of online and mobile phone-delivered interventions for SMI, potential predictors of acceptability, and qualitative themes relating to acceptability. This review also identified that acceptability is far more complex than just module completion rates and intervention use, suggesting the need for continued service user involvement and the inclusion of participant satisfaction ratings and qualitative interviews to measure acceptability.

The hypothetical acceptability of online and mobile phone-delivered interventions for SMI generally varied between studies. However, the relatively low levels of participant interest in online and mobile phone-delivered interventions evident in some of the studies indicate that some people with SMI may be negatively predisposed toward these delivery formats. This contrasts greatly with the relatively high levels of actual acceptability observed in the included studies.

The results from this review also indicated that hypothetical acceptability was higher for interventions delivered via mobile phones than for online formats. It is, however, important to consider these findings in the context of intervention types proposed. Specifically, studies measuring hypothetical acceptability primarily examined participant interest in using mobile phone and online resources to communicate with health care providers and receive appointment and medication reminders. Due to the transportable and immediately accessible nature of mobile phones, it is unsurprising that participants stated a preference for mobile phone delivery over online. However, the delivery preferences of more complex, time-consuming, and interactive interventions such as cognitive behavioral therapy and psychoeducation remains unknown. Many people are now able to access online content with relative ease on their mobile phones; therefore, it is questionable whether the Internet and mobile phones can be viewed as separate methods of intervention delivery.

The findings also revealed that few studies investigated whether demographic and clinical characteristics were predictors of acceptability. The studies that did investigate the influence of demographic and clinical characteristics on acceptability reported no significant relationships [42,45,54,60,63,75,76,86]; however, a few studies did report a significant relationship [75,76,78,79], or an association at the 10% level [42,86]. In addition, hypothetical acceptability was higher in studies where the mean age of participants was <35 years. The varied findings and limited number of studies prevent us from drawing overall conclusions, and further research is warranted to investigate whether demographic and clinical characteristics predict acceptability.

The review findings also indicated that actual acceptability was higher for participants who were offered remote online support than for those who were not supported [75,76,81]. While it is still too early to know whether acceptability is higher in supported interventions than in unsupported interventions, our findings indicate that the provision of remote support is likely to predict acceptability. While we acknowledge that one of the key advantages of online and mobile phone-delivered interventions is the potential reduction in the cost of trained clinicians, these findings suggest that remote support could be offered to help increase the acceptability of these approaches.

We were able to compare hypothetical acceptability across different geographical areas between 2 studies [34,40,43] (personal communication with D Ben-Zeev, May 2015). Participants in the United States were more interested in health care provider check-ins and appointment and medication reminders than were participants in India. However, interest in receiving psychoeducation and service information was higher for participants in India than for those in the United States. The authors noted that differences between participant interest may reflect the increased availability of mental health information resources already available in the United States [43]. Due to the notable increase in Internet and mobile phone access in developing countries and papers reporting the potential benefits of online and mobile phone-delivered interventions in these nations, research in this area will likely increase at a fast pace over the coming years [86-89].

Key common themes identified in the qualitative studies revealed that some participants found the safety and privacy of online and mobile phone-delivered interventions for SMI acceptable [48,49], while others were concerned that confidentiality may be compromised [54,56,58,61]. Many participants felt that online and mobile phone-delivered interventions needed interactive components to increase acceptability and were generally positive about the provision of online peer support [48,49,51,55,82,84]. Based on participant feedback, it is advisable for researchers to incorporate interactive features and social networking components within online and mobile phone-delivered interventions for SMI. A few concerns about technology literacy and technical issues were reported; therefore, researchers should ensure that participants are comfortable with the chosen format for delivery and that the delivery method functions well [54,55,58,61]. The qualitative studies also revealed participant concerns that some people with SMI may struggle to engage with online and mobile phone-delivered interventions while experiencing acute symptoms [56,82]. Researchers should be mindful about the potential influence of psychological state on acceptability across the phases of illness.

### Strengths and Limitations

This review had several notable strengths. First, the range of databases we searched and the list of search terms we created were comprehensive, which ensured that we obtained eligible studies in the field. Second, the review included studies with quantitative, qualitative, and mixed-methods designs, thus enabling a broad and in-depth analysis of the current work in the field. Third, studies were coextracted on the abstract level by a researcher independent of the research team to ensure eligibility criteria were accurate. In addition, our extraction of the data was systematic and we contacted authors if we required any further information.

Findings from the studies in this review should be considered in the context of some limitations. Many of the studies reviewed measured actual acceptability through module completion rates and intervention use. The sole use of these measures of acceptability is problematic due to the potential influence of other factors, for example, the number of modules available to complete, the intervention duration, financial incentives for high completion rates, technical issues with the interventions delivered, participants’ engaging in other activities while logged in, and time pressures preventing engagement. Therefore, module completion rates and intervention use are unlikely to be robust direct measures of acceptability. Across studies, module completion rates and intervention use were also reported in different ways, for example, some reported the average number of modules completed, while others reported the average duration participants spent accessing an intervention. It is impossible to determine what value constitutes an “acceptable” intervention without a universal measurement applied. Future research is needed to develop more accurate ways to assess acceptability.

The majority of the studies we reviewed that measured hypothetical acceptability asked participants about their general interest and willingness to use interventions delivered online and via mobile phones. However, participants were not asked about their interest in receiving these interventions online or via mobile phones in comparison with, or in addition to, face-to-face delivery. Had these questions been phrased differently, overall hypothetical acceptability may have been very different. The review findings also highlight the very limited amount of relevant information regarding predictors of acceptability being reported in studies. We could not draw conclusions about the influence of demographic and clinical characteristics on both hypothetical and actual acceptability.

Issues relating to the heterogeneity of approaches for participant recruitment are an important consideration for the recruitment strategies in the field. Specifically, it could be argued that online recruitment methods may bias the sample toward favorable attitudes toward online and mobile phone-delivered interventions, thus increasing levels of acceptability. However, potential bias toward the acceptability of interventions delivered via these modalities may also be prevalent in more traditional routes of recruitment (ie, through service providers and clinicians). While different recruitment strategies may attract different samples within the population, broadening intervention choice and examining acceptability remain important considerations within the samples identified, regardless of the recruitment method that is used.

There were also some limitations to the method of analysis we used. First, the review excluded papers published in a language other than English. Second, some studies included both participants with SMI and participants with other mental health problems such as depression. To combat this issue, we excluded studies if ≤50% of the total study sample had SMI, so we may have missed relevant findings for those who did experience SMI.

### Implications and Future Research

Although this review highlights the relatively high acceptability of online and mobile phone-delivered interventions for SMI, it also demonstrates the complex nature of acceptability and the need for continued focus in this area. A recent systematic review concluded that, rather than concentrating on acceptability, researchers should instead investigate whether online and mobile phone-delivered interventions are effective [37]. While efficacy is undoubtedly important, we argue that acceptability remains equally important because, ultimately, if an intervention is not acceptable to service users and relevant stakeholders, people are unlikely to engage with the approach, thereby directly affecting efficacy. Based on the findings in this review, we recommend that research groups measure hypothetical and actual acceptability of online and mobile phone-delivered interventions, with an increased focus on the factors that could influence acceptability.

There were different reporting styles evident in studies detailing intervention usage, module completion rates, and session attendance. For example, some studies reported the overall proportion of participants who used the intervention over the whole study period, while others reported proportions over specific weeks during the study period. Therefore, it was not possible to investigate how the acceptability of online and mobile phone-delivered interventions changed over time. Future research should explore and report how intervention usage, module completion rates, and session attendance change throughout intervention delivery to determine whether acceptability of interventions delivered via these modalities changes across time points.

Module completion rates and intervention use may be indirectly related to actual acceptability; however, participant satisfaction ratings and qualitative views provide rich data about acceptability. These rich data can be used to develop and refine online and mobile phone-delivered interventions in order to improve the overall acceptability for people with SMI. The need to use qualitative analysis to inform the design and development of interventions delivered online and via mobile phones has been recognized in protocols for future studies in the field [90-92]. It is recommended that, if researchers choose to measure acceptability through module completion rates and intervention use, satisfaction ratings and qualitative interviews be conducted to obtain the rich information needed to identify intervention acceptability. In addition, the use of qualitative interviews examining what participants feel they have actually gained from an intervention may help to highlight the specific areas of their lives that they feel may have been improved by participating in the intervention.

This review also found that hypothetical acceptability tended to be low or varied, while actual acceptability tended to be high, indicating that people with SMI may be initially reluctant to engage with online and mobile phone-delivered interventions. However, hypothetical acceptability results are largely limited and not necessarily directly comparable with the interventions being delivered. Issues with comparing hypothetical versus actual acceptability are primarily due to discrepancies in the types of interventions being explored. Specifically, studies investigating hypothetical acceptability investigated interest in using the Internet or mobile phones to facilitate health care provider contact and medication and appointment reminders, whereas studies investigating actual acceptability tended to implement more complex interventions such as cognitive behavioral therapy and psychoeducation. It is also likely that the sample of people who were asked about actual acceptability were already hypothetically open to the idea of receiving online and mobile phone-delivered interventions. We therefore suggest that researchers measure acceptability both before (hypothetical acceptability) and after (actual acceptability) an intervention is delivered. These measurements will aid the comparison of hypothetical versus actual acceptability without the limitations associated with separate samples.

Crucially, this review showed that the majority of the studies reviewed recruited participants who were already in contact with mental health services. One of the potential advantages of the Internet and mobile phones is that they could improve access to evidence-based interventions for people who are not receiving support but who need or want it. Therefore, it is important to investigate whether people who could potentially benefit the most from online and mobile phone-delivered interventions actually find these delivery formats acceptable.

Clearly, interventions delivered online and via mobile phones do have their place in the provision of self-care for people with SMI. However, research has yet to identify predictors of acceptability and whether people who are not engaged with services also find online and mobile phone-delivered approaches acceptable. The measurement of both hypothetical and actual acceptability in future studies would enable the investigation of the impact of prior expectations on acceptability and potential changes in acceptability after access. In order to obtain rich data about acceptability, we recommend the measurement of satisfaction ratings and participant views and the continued involvement of service users throughout all aspects of intervention development and delivery.

## Appendix

Multimedia Appendix 1 Characteristics of studies that measured the hypothetical acceptability of online and mobile phone-delivered interventions for severe mental health problems.

Multimedia Appendix 2 Characteristics of studies that measured the actual acceptability of online and mobile phone-delivered interventions for SMI.

Multimedia Appendix 3 Results and predictors relating to the hypothetical acceptability of online and mobile phone-delivered interventions for people with severe mental health problems.

Multimedia Appendix 4 Results and predictors relating to the actual acceptability of online and mobile phone-delivered interventions for people with SMI measured by intervention use, module completion rates, and participant satisfaction.

Multimedia Appendix 5 Participant views and example quotes from studies that measured the acceptability of online and mobile phone-delivered interventions for people with SMI.

## Abbreviations

IAPT: Improving Access to Psychological Therapies
NHS: National Health Service
PRISMA: Preferred Reporting Items for Systematic Reviews and Meta-Analyses
SMI: severe mental health problems

## References

[1] Parabiaghi A, Bonetto C, Ruggeri M, Lasalvia A, Leese M (2006). Severe and persistent mental illness: a useful definition for prioritizing community-based mental health service interventions. Soc Psychiatry Psychiatr Epidemiol.

[2] Ruggeri M, Leese M, Thornicroft G, Bisoffi G, Tansella M (2000). Definition and prevalence of severe and persistent mental illness. Br J Psychiatry.

[3] Cooke A (2014). Understanding Psychosis and Schizophrenia: Why People Sometimes Hear Voices, Believe Things That Others Find Strange, or Appear Out of Touch With Reality, and What Can Help.

[4] Improving Access to Psychological Therapies (2015). Severe Mental Illness.

[5] National Institute for Health and Care Excellence (2009). Borderline Personality Disorder: Treatment and Management.

[6] National Institute for Health and Care Excellence (2014). Psychosis and Schizophrenia in Adults: Treatment and Management.

[7] National Institute for Health and Care Excellence (2014). Bipolar Disorder: The Assessment and Management of Bipolar Disorder in Adults, Children and Young People in Primary and Secondary Care.

[8] Clement S, Schauman O, Graham T, Maggioni F, Evans-Lacko S, Bezborodovs N, Morgan C, Rüsch N, Brown JSL, Thornicroft G (2015). What is the impact of mental health-related stigma on help-seeking? A systematic review of quantitative and qualitative studies. Psychol Med.

[9] Morriss R (2008). Implementing clinical guidelines for bipolar disorder. Psychol Psychother.

[10] Berry K, Haddock G (2008). The implementation of the NICE guidelines for schizophrenia: barriers to the implementation of psychological interventions and recommendations for the future. Psychol Psychother.

[11] Mind (2013). We Still Need to Talk: A Report on Access to Talking Therapies.

[12] (2015). The Five Year Forward View Mental Health Taskforce: Public Engagement Findings.

[13] Cotter AP, Durant N, Agne AA, Cherrington AL (2014). Internet interventions to support lifestyle modification for diabetes management: a systematic review of the evidence. J Diabetes Complications.

[14] Holtz B, Lauckner C (2012). Diabetes management via mobile phones: a systematic review. Telemed J E Health.

[15] Northouse L, Schafenacker A, Barr KL, Katapodi M, Yoon H, Brittain K, Song L, Ronis DL, An L (2014). A tailored Web-based psychoeducational intervention for cancer patients and their family caregivers. Cancer Nurs.

[16] Park LG, Howie-Esquivel J, Chung ML, Dracup K (2014). A text messaging intervention to promote medication adherence for patients with coronary heart disease: a randomized controlled trial. Patient Educ Couns.

[17] Bundy C, Pinder B, Bucci S, Reeves D, Griffiths CEM, Tarrier N (2013). A novel, web-based, psychological intervention for people with psoriasis: the electronic Targeted Intervention for Psoriasis (eTIPs) study. Br J Dermatol.

[18] Civljak M, Stead LF, Hartmann-Boyce J, Sheikh A, Car J (2013). Internet-based interventions for smoking cessation. Cochrane Database Syst Rev.

[19] Whittaker R, McRobbie H, Bullen C, Borland R, Rodgers A, Gu Y (2012). Mobile phone-based interventions for smoking cessation. Cochrane Database Syst Rev.

[20] Hamel LM, Robbins LB, Wilbur J (2011). Computer- and web-based interventions to increase preadolescent and adolescent physical activity: a systematic review. J Adv Nurs.

[21] Stephens J, Allen J (2013). Mobile phone interventions to increase physical activity and reduce weight: a systematic review. J Cardiovasc Nurs.

[22] Lal S, Adair CE (2014). E-mental health: a rapid review of the literature. Psychiatr Serv.

[23] Hollis C, Morriss R, Martin J, Amani S, Cotton R, Denis M, Lewis S (2015). Technological innovations in mental healthcare: harnessing the digital revolution. Br J Psychiatry.

[24] Ebert DD, Zarski AC, Christensen H, Stikkelbroek Y, Cuijpers P, Berking M, Riper H (2015). Internet and computer-based cognitive behavioral therapy for anxiety and depression in youth: a meta-analysis of randomized controlled outcome trials. PLoS One.

[25] Richards D, Richardson T (2012). Computer-based psychological treatments for depression: a systematic review and meta-analysis. Clin Psychol Rev.

[26] Schlegl S, Bürger C, Schmidt L, Herbst N, Voderholzer U (2015). The potential of technology-based psychological interventions for anorexia and bulimia nervosa: a systematic review and recommendations for future research. J Med Internet Res.

[27] Marsch LA, Carroll KM, Kiluk BD (2014). Technology-based interventions for the treatment and recovery management of substance use disorders: a JSAT special issue. J Subst Abuse Treat.

[28] Alvarez-Jimenez M, Alcazar-Corcoles MA, González-Blanch C, Bendall S, McGorry PD, Gleeson JF (2014). Online, social media and mobile technologies for psychosis treatment: a systematic review on novel user-led interventions. Schizophr Res.

[29] van der Krieke L, Wunderink L, Emerencia AC, de Jonge P, Sytema S (2014). E-mental health self-management for psychotic disorders: state of the art and future perspectives. Psychiatr Serv.

[30] Hidalgo-Mazzei D, Mateu A, Reinares M, Matic A, Vieta E, Colom F (2015). Internet-based psychological interventions for bipolar disorder: Review of the present and insights into the future. J Affect Disord.

[31] Rizvi SL, Dimeff LA, Skutch J, Carroll D, Linehan MM (2011). A pilot study of the DBT coach: an interactive mobile phone application for individuals with borderline personality disorder and substance use disorder. Behav Ther.

[32] Trefflich F, Kalckreuth S, Mergl R, Rummel-Kluge C (2015). Psychiatric patients' internet use corresponds to the internet use of the general public. Psychiatry Res.

[33] Carras MC, Mojtabai R, Furr-Holden CD, Eaton W, Cullen BA (2014). Use of mobile phones, computers and internet among clients of an inner-city community psychiatric clinic. J Psychiatr Pract.

[34] Ben-Zeev D, Davis KE, Kaiser S, Krzsos I, Drake RE (2013). Mobile technologies among people with serious mental illness: opportunities for future services. Adm Policy Ment Health.

[35] Firth J, Cotter J, Torous J, Bucci S, Firth JA, Yung AR (2016). Mobile Phone Ownership and Endorsement of “mHealth” Among People With Psychosis: A Meta-analysis of Cross-sectional Studies. Schizophr Bull.

[36] Glick G, Druss B, Pina J, Lally C, Conde M (2015). Use of mobile technology in a community mental health setting. J Telemed Telecare.

[37] Naslund JA, Marsch LA, McHugo GJ, Bartels SJ (2015). Emerging mHealth and eHealth interventions for serious mental illness: a review of the literature. J Ment Health.

[38] Firth J, Torous J (2015). Smartphone apps for schizophrenia: a systematic review. JMIR Mhealth Uhealth.

[39] Moher D, Liberati A, Tetzlaff J, Altman DG (2009). Preferred reporting items for systematic reviews and meta-analyses: the PRISMA statement. BMJ.

[40] Ben-Zeev D, Kaiser SM, Brenner CJ, Begale M, Duffecy J, Mohr DC (2013). Development and usability testing of FOCUS: a smartphone system for self-management of schizophrenia. Psychiatr Rehabil J.

[41] Birnbaum ML, Rizvi AF, Correll CU, Kane JM (2015). Role of social media and the Internet in pathways to care for adolescents and young adults with psychotic disorders and non-psychotic mood disorders. Early Interv Psychiatry.

[42] Bogart K, Wong SK, Lewis C, Akenzua A, Hayes D, Prountzos A, Okocha CI, Kravariti E (2014). Mobile phone text message reminders of antipsychotic medication: is it time and who should receive them? A cross-sectional trust-wide survey of psychiatric inpatients. BMC Psychiatry.

[43] Jain N, Singh H, Koolwal GD, Kumar S, Gupta A (2015). Opportunities and barriers in service delivery through mobile phones (mHealth) for severe mental illnesses in Rajasthan, India: a multi-site study. Asian J Psychiatr.

[44] Lal S, Dell'Elce J, Tucci N, Fuhrer R, Tamblyn R, Malla A (2015). Preferences of young adults with first-episode psychosis for receiving specialized mental health services using technology: a survey study. JMIR Mental Health.

[45] Miller BJ, Stewart A, Schrimsher J, Peeples D, Buckley PF (2015). How connected are people with schizophrenia? Cell phone, computer, email, and social media use. Psychiatry Res.

[46] Sanghara H, Kravariti E, Jakobsen H, Okocha C (2010). Using short message services in mental health services: assessing feasibility. Mental Health Rev J.

[47] Alvarez-Jimenez M, Bendall S, Lederman R, Wadley G, Chinnery G, Vargas S, Larkin M, Killackey E, McGorry PD, Gleeson JF (2013). On the HORYZON: moderated online social therapy for long-term recovery in first episode psychosis. Schizophr Res.

[48] Gleeson JF, Lederman R, Wadley G, Bendall S, McGorry PD, Alvarez-Jimenez M (2014). Safety and privacy outcomes from a moderated online social therapy for young people with first-episode psychosis. Psychiatr Serv.

[49] Lederman R, Wadley G, Gleeson J, Bendall S, Alvarez-Jimenez M (2014). Moderated online social therapy: designing and evaluating technology for mental health. ACM Trans Comput Hum Interact.

[50] Kuosmanen L, Välimäki M, Joffe G, Pitkänen A, Hätönen H, Patel A, Knapp M (2009). The effectiveness of technology-based patient education on self-reported deprivation of liberty among people with severe mental illness: a randomized controlled trial. Nord J Psychiatry.

[51] Hätönen H, Suhonen R, Warro H, Pitkänen A, Välimäki M (2010). Patients' perceptions of patient education on psychiatric inpatient wards: a qualitative study. J Psychiatr Ment Health Nurs.

[52] Kuosmanen L, Jakobsson T, Hyttinen J, Koivunen M, Välimäki M (2010). Usability evaluation of a web-based patient information system for individuals with severe mental health problems. J Adv Nurs.

[53] Pitkänen A, Välimäki M, Kuosmanen L, Katajisto J, Koivunen M, Hätönen H, Patel A, Knapp M (2012). Patient education methods to support quality of life and functional ability among patients with schizophrenia: a randomised clinical trial. Qual Life Res.

[54] Anttila M, Välimäki M, Hätönen H, Luukkaala T, Kaila M (2012). Use of web-based patient education sessions on psychiatric wards. Int J Med Inform.

[55] Laine A, Anttila M, Välimäki M (2016). Modification of an internet-based patient education program for adults with schizophrenia spectrum disorder to suit adolescents with psychosis. Inform Health Soc Care.

[56] Barnes E, Simpson S, Griffiths E, Hood K, Craddock N, Smith DJ (2011). Developing an online psychoeducation package for bipolar disorder. J Ment Health.

[57] Smith DJ, Griffiths E, Poole R, di Florio A, Barnes E, Kelly MJ, Craddock N, Hood K, Simpson S (2011). Beating Bipolar: exploratory trial of a novel Internet-based psychoeducational treatment for bipolar disorder. Bipolar Disord.

[58] Poole R, Simpson SA, Smith DJ (2012). Internet-based psychoeducation for bipolar disorder: a qualitative analysis of feasibility, acceptability and impact. BMC Psychiatry.

[59] Barnes CW, Hadzi-Pavlovic D, Wilhelm K, Mitchell PB (2015). A web-based preventive intervention program for bipolar disorder: outcome of a 12-months randomized controlled trial. J Affect Disord.

[60] Ben-Zeev D, Brenner CJ, Begale M, Duffecy J, Mohr DC, Mueser KT (2014). Feasibility, acceptability, and preliminary efficacy of a smartphone intervention for schizophrenia. Schizophr Bull.

[61] de Leeuw JRJ, van Splunteren P, Boerema I (2012). Personal control in rehabilitation: An internet platform for patients with schizophrenia and their caregivers. Open J Psychiatry.

[62] Depp CA, Mausbach B, Granholm E, Cardenas V, Ben-Zeev D, Patterson TL, Lebowitz BD, Jeste DV (2010). Mobile interventions for severe mental illness: design and preliminary data from three approaches. J Nerv Ment Dis.

[63] Depp CA, Ceglowski J, Wang VC, Yaghouti F, Mausbach BT, Thompson WK, Granholm EL (2015). Augmenting psychoeducation with a mobile intervention for bipolar disorder: a randomized controlled trial. J Affect Disord.

[64] Granholm E, Ben-Zeev D, Link PC, Bradshaw KR, Holden JL (2012). Mobile Assessment and Treatment for Schizophrenia (MATS): a pilot trial of an interactive text-messaging intervention for medication adherence, socialization, and auditory hallucinations. Schizophr Bull.

[65] Forchuk C, Donelle L, Ethridge P, Warner L (2015). Client perceptions of the Mental Health Engagement Network: a secondary analysis of an intervention using smartphones and desktop devices for individuals experiencing mood or psychotic disorders in Canada. JMIR Ment Health.

[66] Gottlieb JD, Romeo KH, Penn DL, Mueser KT, Chiko BP (2013). Web-based cognitive-behavioral therapy for auditory hallucinations in persons with psychosis: a pilot study. Schizophr Res.

[67] Holländare F, Eriksson A, Lövgren L, Humble MB, Boersma K (2015). Internet-based cognitive behavioral therapy for residual symptoms in bipolar disorder type II: A single-subject design pilot study. JMIR Res Protoc.

[68] Kane JM, Perlis RH, DiCarlo LA, Au-Yeung K, Duong J, Petrides G (2013). First experience with a wireless system incorporating physiologic assessments and direct confirmation of digital tablet ingestions in ambulatory patients with schizophrenia or bipolar disorder. J Clin Psychiatry.

[69] Latalova K, Prasko J, Kamaradova D, Jelenova D, Ociskova M, Sedlackova Z (2014). Internet psychoeducation for bipolar affective disorder: basis for preparation and first experiences. Psychiatr Q.

[70] Lauder S, Chester A, Castle D, Dodd S, Berk L, Klein B, Austin D, Gilbert M, Chamberlain JA, Murray G, White C, Piterman L, Berk M (2013). Development of an online intervention for bipolar disorder. www.moodswings.net.au. Psychol Health Med.

[71] Lauder S, Chester A, Castle D, Dodd S, Gliddon E, Berk L, Chamberlain J, Klein B, Gilbert M, Austin DW, Berk M (2015). A randomized head to head trial of MoodSwings.net.au: an Internet based self-help program for bipolar disorder. J Affect Disord.

[72] Lieberman DZ, Swayze S, Goodwin FK (2011). An automated Internet application to help patients with bipolar disorder track social rhythm stabilization. Psychiatr Serv.

[73] Miklowitz DJ, Price J, Holmes EA, Rendell J, Bell S, Budge K, Christensen J, Wallace J, Simon J, Armstrong NM, McPeake L, Goodwin GM, Geddes JR (2012). Facilitated Integrated Mood Management for adults with bipolar disorder. Bipolar Disord.

[74] Murray G, Leitan ND, Berk M, Thomas N, Michalak E, Berk L, Johnson SL, Jones S, Perich T, Allen NB, Kyrios M (2015). Online mindfulness-based intervention for late-stage bipolar disorder: pilot evidence for feasibility and effectiveness. J Affect Disord.

[75] Nicholas J, Proudfoot J, Parker G, Gillis I, Burckhardt R, Manicavasagar V, Smith M (2010). The ins and outs of an online bipolar education program: a study of program attrition. J Med Internet Res.

[76] Proudfoot J, Parker G, Manicavasagar V, Hadzi-Pavlovic D, Whitton A, Nicholas J, Smith M, Burckhardt R (2012). Effects of adjunctive peer support on perceptions of illness control and understanding in an online psychoeducation program for bipolar disorder: a randomised controlled trial. J Affect Disord.

[77] Pijnenborg GHM, Withaar FK, Brouwer WH, Timmerman ME, van den Bosch J, Evans JJ (2010). The efficacy of SMS text messages to compensate for the effects of cognitive impairments in schizophrenia. Br J Clin Psychol.

[78] Rotondi AJ, Haas GL, Anderson CM, Newhill CE, Spring MB, Ganguli R, Gardner WB, Rosenstock JB (2005). A clinical trial to test the feasibility of a telehealth psychoeducational intervention for persons with schizophrenia and their families: intervention and 3-month findings. Rehabil Psychol.

[79] Rotondi AJ, Anderson CM, Haas GL, Eack SM, Spring MB, Ganguli R, Newhill C, Rosenstock J (2010). Web-based psychoeducational intervention for persons with schizophrenia and their supporters: one-year outcomes. Psychiatr Serv.

[80] Rotondi AJ, Eack SM, Hanusa BH, Spring MB, Haas GL (2015). Critical design elements of e-health applications for users with severe mental illness: singular focus, simple architecture, prominent contents, explicit navigation, and inclusive hyperlinks. Schizophr Bull.

[81] Simon GE, Ludman EJ, Goodale LC, Dykstra DM, Stone E, Cutsogeorge D, Operskalski B, Savarino J, Pabiniak C (2011). An online recovery plan program: can peer coaching increase participation?. Psychiatr Serv.

[82] Todd NJ, Jones SH, Lobban FA (2013). What do service users with bipolar disorder want from a web-based self-management intervention? A qualitative focus group study. Clin Psychol Psychother.

[83] Todd NJ, Jones SH, Hart A, Lobban FA (2014). A web-based self-management intervention for bipolar disorder 'living with bipolar': a feasibility randomised controlled trial. J Affect Disord.

[84] van der Krieke L, Emerencia AC, Aiello M, Sytema S (2012). Usability evaluation of a web-based support system for people with a schizophrenia diagnosis. J Med Internet Res.

[85] van der Krieke L, Emerencia AC, Boonstra N, Wunderink L, de Jonge P, Sytema S (2013). A web-based tool to support shared decision making for people with a psychotic disorder: randomized controlled trial and process evaluation. J Med Internet Res.

[86] Wenze SJ, Armey MF, Miller IW (2014). Feasibility and acceptability of a mobile intervention to improve treatment adherence in bipolar disorder: A pilot study. Behav Modif.

[87] Pew Research Center (2015). Communications Technology in Emerging and Developing Nations.

[88] Brian RM, Ben-Zeev D (2014). Mobile health (mHealth) for mental health in Asia: objectives, strategies, and limitations. Asian J Psychiatr.

[89] Farrington C, Aristidou A, Ruggeri K (2014). mHealth and global mental health: still waiting for the mH2 wedding?. Global Health.

[90] Bucci S, Barrowclough C, Ainsworth J, Morris R, Berry K, Machin M, Emsley R, Lewis S, Edge D, Buchan I, Haddock G (2015). Using mobile technology to deliver a cognitive behaviour therapy-informed intervention in early psychosis (Actissist): study protocol for a randomised controlled trial. Trials.

[91] Lobban F, Dodd AL, Dagnan D, Diggle PJ, Griffiths M, Hollingsworth B, Knowles D, Long R, Mallinson S, Morriss RM, Parker R, Sawczuk AP, Jones S (2015). Feasibility and acceptability of web-based enhanced relapse prevention for bipolar disorder (ERPonline): trial protocol. Contemp Clin Trials.

[92] Hidalgo-Mazzei D, Mateu A, Reinares M, Undurraga J, Bonnín Cdel M, Sánchez-Moreno J, Vieta E, Colom F (2015). Self-monitoring and psychoeducation in bipolar patients with a smart-phone application (SIMPLe) project: design, development and studies protocols. BMC Psychiatry.

