# Supplementary material for: Acceptability of Interventions Delivered Online and Through Mobile Phones for People Who Experience Severe Mental Health Problems: A Systematic Review
Source: J Med Internet Res. 2016 May 31;18(5):e121. doi: 10.2196/jmir.5250 (PMC4908305; doi:10.2196/jmir.5250)
Supplement: Multimedia Appendix 1 [file jmir_v18i5e121_app1.pdf]

Table 1. Characteristics of studies that measured the hypothetical acceptability of online and mobile phone-delivered interventions for severe mental health problems.

Author(s), year, location	Diagnosis	Proposed intervention and delivery method	Study design and recruitment setting	Sample size (N)	Mean (SD) age, years	Sex (% (male)
Ben-Zeev et al[a,b] [34,40], D Ben-Zeev, PhD, written communication, May 2015[c]; 2013, US	Clinician-confirmed Schizophrenia, schizoaffective disorder, or bipolar disorder	Mobile and online: medication or appointment reminders; check-ins with provider; information about services	Survey; Thresholds (psychiatric rehabilitation agency)	Total: 1237; schizophrenia or schizoaffective disorder: 904; bipolar disorder: 333	Overall sample (including other diagnoses): 46 (12)	Overall sample (including other diagnoses): 61%
Birnbaum et al[a] [41], A Rizvi, MA, written communication, May 2015[c]; 2015, US	Clinician-confirmed: psychotic spectrum disorders or bipolar disorder	Online (social media): clinicians approaching during active symptom emergence; obtaining help/advice via social media	Semistructured interviews; Zucker Hillside Hospital (adult and adolescent inpatient units and outpatient departments)	Total: 51; psychotic disorders: 40; bipolar disorder: 11	Psychotic disorder: 18.75; bipolar disorder: 17.91	Psychotic disorder: 65; bipolar disorder: 27.27
Bogart et al [42]; 2014, UK	Clinician-confirmed psychotic disorders	Mobile: medication reminders	Survey; Oxleas National Health Service Foundation Trust inpatient units	85	NR[d]	55
Jain et al [43]; 2015, India	Clinician-confirmed: schizophrenia, bipolar disorder, or psychosis NOS[e]	Mobile and online: medication or appointment reminders; check-ins with provider; information about services; telephonic follow-ups; helpline for crisis	Survey; 3 free tertiary care hospitals providing inpatient and outpatient care	Total: 201; schizophrenia: 106; bipolar disorder: 65; psychosis NOS: 30	Median: 40	83

Lal et al [44]; 2015, Canada	Clinician-confirmed FEP[f]	Mobile and online: social media for information and support; medication or appointment reminders/scheduling; information; decision-making tools; contact with provider; coping skills	Survey; 2 specialized early intervention programs for FEP	67	25.6 (5.1)	76.1
Miller et al [45]; 2015, US	Clinician-confirmed schizophrenia or schizoaffective disorder	Mobile and online: medication or appointment reminders; contact with doctor	Survey; Georgia Regents University Inpatient Psychiatry Unit or Adult Psychiatry Outpatient Clinic	80	41 (13)	51
Sanghara et al[a] [46]; 2010, UK	Clinician-confirmed psychotic disorders	Mobile: willingness to receive text messages from the Trust	Survey; Oxleas National Health Service Foundation Trust inpatient units	100	NR	Overall sample (including other diagnoses): 59.57

[a]Study also included participants with different diagnoses; results for these participants are not reported in this review.

[b]Study included data from caregivers or clinicians, or both; results from these participants are not reported in this review.

[c]Study results presented in multiple papers or provided through personal communication.

[d]NR: not reported.

[e]NOS: not otherwise specified.

[f]First episode psychosis.
