# Supplementary material for: Acceptability of Interventions Delivered Online and Through Mobile Phones for People Who Experience Severe Mental Health Problems: A Systematic Review
Source: J Med Internet Res. 2016 May 31;18(5):e121. doi: 10.2196/jmir.5250 (PMC4908305; doi:10.2196/jmir.5250)
Supplement: Multimedia Appendix 2 [file jmir_v18i5e121_app2.pdf]

Table 2. Characteristics of studies that measured the actual acceptability of online and mobile phone-delivered interventions for SMI.

Project, location, diagnosis, and intervention	Author(s) & year	Study design	Recruitment setting	Intervention duration and module/ session numbers	Sample size (n)	Mean (SD) age, years	Sex (% male)
HORYZONS<br><br>Australia<br><br>Clinician confirmed:<br>FEP or mood disorder with psychotic features<br><br>Online interactive psychosocial interventions & moderated social networking.	Alvarez-Jimenez et al [47]<br>2013	Uncontrolled pilot study	Early Psychosis Prevention and Intervention Centre (EPPIC, Melbourne)	4 weeks<br><br>7 modules	20	20.3 (2.7)	50
	Gleeson et al [48]<br><br>2013	Qualitative analysis of uncontrolled pilot study	Early Psychosis Prevention and Intervention Centre (EPPIC, Melbourne)	4 weeks<br><br>7 modules	20	20.3 (2.7)	50
	Lederman, Wadley, Gleeson, Bendall, & Alvarez-Jimenez [49]<br><br>2014	Qualitative analysis of uncontrolled pilot study	Early Psychosis Prevention and Intervention Centre (EPPIC, Melbourne)	4 weeks<br><br>7 modules	20	20.3 (2.7)	50
Mental.Net<br><br>Finland<br><br>Clinician confirmed:<br>Schizophrenia spectrum psychosis<br><br>Online nurse-	Kuosmanen et al [50]<br><br>2009	Randomized Controlled Trial (RCT):<br><br>3 conditions:<br>IT-based education (IT); conventional education (CE); standard care (SC)	9 acute inpatient wards in 2 psychiatric hospitals	'About' a month<br><br>5 sessions	Total:<br>311<br><br>IT:<br>100<br><br>CE:<br>106<br><br>SC:<br>105	IT:<br>37.3 (12.4)<br><br>CE:<br>39.1 (12.6)<br><br>SC:<br>37.9 (12.8)	IT:<br>61<br><br>CE:<br>61<br><br>SC<br>55

supported patient education							
	Hätönen, Suhonen, Warro, Pitkänen, & Välimäki [51] 2010	Qualitative analysis of RCT:<br><br>3 conditions: IT-based education; conventional education; standard care	9 acute inpatient wards in 2 psychiatric hospitals	'About' a month<br><br>5 sessions	Total: 16<br><br>IT: 6<br><br>CE: 6<br><br>SC: 4	35.7 (11.92)	68.75
	Kuosmanen, Jakobsson, Hyttinen, Koivunen, & Välimäki[b] [52] 2010	Uncontrolled pilot study	2 local service user mental health associations	Not applicable (NA) (usability testing)	21	Not reported (NR)	48
	Pitkänen et al [53] 2012	Further analysis of RCT:<br><br>3 conditions: IT-based education; conventional education; standard care	9 acute inpatient wards in 2 psychiatric hospitals	'About' a month<br><br>5 sessions	Total: 311<br><br>IT: 100<br><br>CE: 106<br><br>SC: 105	IT: 37.3 (12.4)<br><br>CE: 39.1 (12.6)<br><br>SC 37.9 (12.8)	IT: 61<br><br>CE: 61<br><br>SC: 51
	Anttila, Välimäki, Hätönen, Luukkaala, Kaila [54] 2012	Further analysis of RCT:<br><br>3 conditions: IT-based education; conventional education;	9 acute inpatient wards in 2 psychiatric hospitals	'About' a month<br><br>6 sessions	100	37.2 (12.2)	62.4

		standard care<br><br>(Reports on the IT education group)					
	Laine, Anttila, & Välimäki[b] [55] 2015	Phase 1: Qualitative needs assessment;<br><br>Phase 2: Preliminary evaluation feedback	Psychiatric rehabilitation unit for adolescents in 1 hospital	N/A (felt needs and initial thoughts)	Phase 1: 6<br><br>Phase 2: 2	NR	50
Beating Bipolar<br><br>UK<br><br>Clinician confirmed: Bipolar disorder<br><br>Online interactive psychoeducation plus moderated discussion forum	Barnes et al[a,b] [56] 2011	Focus groups	Participants from another part of the study	N/A (focus groups)	8	NR	NR
	Smith et al [57]<br><br>2011	RCT:<br><br>2 conditions: Beating Bipolar plus TAU; TAU only	Primary care practices & community mental health teams. Case files from the Mental Health Research Network Cymru (Wales). Members of the Manic Depression Fellowship in Wales through local group coordinators	4 months<br><br>8 modules	Total: 50<br><br>Beating Bipolar: 24<br><br>TAU: 26	Beating Bipolar: 42.7 (11.4)<br><br>TAU: 44.7 (9.9)	Beating Bipolar: 45.8<br><br>TAU: 30.8
	Poole, Simpson, & Smith [58]	Qualitative analysis of RCT:	Primary care practices & community	4 months<br><br>8 modules	Total: 20	NR	High users: 57.14

	2012	2 conditions:<br>Beating Bipolar plus TAU;<br>TAU only	mental health teams. Case files from the Mental Health Research Network Cymru (Wales). Members of the Manic Depression Fellowship in Wales through local group coordinators		High users:<br>14<br><br>Low users:<br>6		Low users:<br>83.33
Health Steps for Bipolar Disorder<br>Australia<br>Confirmed by clinical questionnaire: Bipolar disorder<br>Online psychoeducation plus CBT & mood monitoring	Barnes, Hadzi-Pavlovic, Wilhelm, & Mitchell [59]<br>2015	RCT:<br><br>2 conditions:<br>Health Steps for Bipolar Disorder;<br>Attention control (AC)	Online recruitment	12 months<br><br>20 sessions	Total:<br>233<br><br>Health Steps:<br>113<br><br>AC:<br>120	Health Steps:<br>40.9<br><br>AC:<br>37.6	Health Steps:<br>26.5<br><br>AC:<br>29.2
FOCUS<br>US<br>Clinician confirmed: Schizophrenia or schizoaffective disorder<br>Mobile (smartphone) assessment prompts & various evidence-based interventions to target psychotic	Ben-Zeev et al[b] [40]<br>2013	Pilot usability testing	NR	N/A (usability testing)	12	45	67
	Ben-Zeev et al [60]<br>2014	Pre-post single-group field trial	Community-based treatment programs	1 month<br><br>3 prompts daily	33	45.9 (8.78)	61

symptoms							
Personal control in Rehabilitation (PCR)<br><br>the Netherlands<br><br>Clinician confirmed:<br>FEP or schizophrenia<br><br>Online patient information, self-management, psychoeducation, and digital contact with other patients and caregivers	de Leeuw, van Splunteren, & Boerema[b] [61] 2012	Uncontrolled pilot study	NR	NR	Main study: 60<br><br>Subsequent focus group: 19	NR	Main study: NR<br><br>Focus group: 73.68
PRISM<br>US<br>Clinician confirmed:<br>Bipolar disorder<br>In-person psychoeducation followed by mobile symptom monitoring plus coping strategies feedback	Depp et al [62]<br><br>2010	Uncontrolled pilot study	NR	2 weeks<br><br>4 assessment prompts daily	10	41 (13.7)	NR
	Depp et al [63]<br><br>2015	RCT:<br><br>2 conditions: PRISM; paper-and-pencil mood charting	Advertisements & posters online & in community residential & drop-in settings, self-help support groups, & outpatient psychiatric clinics	10 weeks<br><br>2 survey prompts daily	PRISM: 41<br><br>Paper-and-pencil: 41	PRISM: 46.9 (11.8)<br><br>Paper-and-pencil: 48.1 (12.9)	PRISM: 46.3<br><br>Paper-and-pencil: 36.6
MATS<br>US<br>Clinician	Depp et al [62]<br><br>2010	Uncontrolled pilot study	NR	12 weeks<br><br>3 assessment	8	NR	NR

confirmed:<br>Schizophrenia or schizoaffective disorder<br>Mobile symptom monitoring plus CBT techniques				prompts daily			
	Granholm, Ben-Zeev, Link, Bradshaw, & Holden [64]<br><br>2012	Uncontrolled pilot study	Outpatient residential & community settings in the Veterans Affairs San Diego Healthcare System & the San Diego County Mental Health System	12 weeks<br><br>12 messages sent each day	Total:<br>55<br><br>Completers:<br>42<br><br>Non completers<br>13	Completers:<br>48.7 (9.1)<br><br>Noncompleters:<br>48.9 (7.9)	Completers:<br>69<br><br>Noncompleters:<br>NR
MHEN<br><br>Canada<br><br>Clinician confirmed:<br>Personality disorder or psychotic disorder<br><br>Mobile and online personal health records, mood monitoring, prompts and reminders, health journals, and secure messaging with mental health professionals	Forchuk, Donelle, Ethridge, & Warner[a] [65], L Warner, MSc, written communication, June 2015; 2015	Secondary analysis of mixed-methods delayed-implementation study	Health care providers from 4 community mental health agencies	18 months	Total:<br>258<br><br>Psychotic disorder:<br>234<br><br>Personality disorder:<br>24	Overall sample (incl. other diagnoses):<br>37.6 (13.8)	Overall sample (incl. other diagnoses):<br>60.7
Coping with Voices<br>US	Gottlieb, Romeo, Penn, Mueser, &	Open pilot study	4 community mental health clinics	10 sessions	21	40.1 (13.63)	62

Clinician confirmed: Psychotic disorder<br>Online logs, exercises, and games based on CBT principles & coping techniques	Chiko [66]<br>2013						
Sweden<br>Clinician confirmed: Bipolar disorder Type II<br>Online CBT with psychoeducation, emotion regulation, sleep quality, cognitive restructuring, long-term goals, & relapse prevention	Holländare, Eriksson, Lövgren, Humble, & Boersma [67]<br>2015	Uncontrolled pilot study	Letters sent to service users on a psychiatry database held by Region örebro County	6 weeks<br><br>6 modules	4	48.75 (14.29)	25
DHFS<br>US<br>Clinician confirmed: Schizophrenia or bipolar disorder<br>Digital sensor to confirm tablet ingestion, wearable sensor, mobile self-reported information, & personalized weekly report	Kane et al [68]<br>2013	Pilot observation study	Referrals & advertisements at the Zucker Hillside Hospital & Massachusetts Hospital	4 weeks	28<br><br>Schizophrenia group:<br>16<br><br>Bipolar disorder group:<br>12	42.8 (12.7)	64.3

Czech Republic<br>Clinician confirmed:<br>Bipolar disorder<br>Online psychoeducation	Latalova et al [69]<br><br>2014	Uncontrolled pilot study	Service users hospitalized in a psychiatric department or attending a day hospital program	12 modules	12	NR	NR
MoodSwings (MS) & MoodSwings Plus (MS-Plus)<br>Australia<br>Confirmed through clinical interview:<br>Bipolar disorder<br>Online psychoeducation, discussion forums (MS), and CBT (MS-Plus)	Lauder et al[b] [70]<br>2013	Uncontrolled pilot study	Referrals from service providers & online	5 core modules; 3 booster modules	12	41.92 (11.16)	16.7
	Lauder et al [71]<br>2015	RCT:<br><br>2 conditions:<br>MS;<br>MS-Plus	Clinician referral, online, & advertising via conferences & consumer & professional forums	12 months<br><br>5 core modules; 3 booster modules	Total:<br>130<br><br>MS:<br>59<br><br>MS-Plus:<br>71	MS:<br>41.35 (9.85)<br><br>MS-Plus:<br>39.87 (11.26)	MS:<br>24<br><br>MS-Plus:<br>27
Unites States<br>Self-reported:<br>Bipolar disorder<br>Online social rhythm therapy, mood charting, and activity planning	Lieberman et al [72]<br>2011	Uncontrolled pilot study	Online advertisements	90 days	64	43 (12.5)	12.7
FIMM<br>US<br>Clinician confirmed:<br>Bipolar disorder<br>In-person psychoeducation followed by online or mobile mood monitoring	Miklowitz et al [73]<br>2012	2 uncontrolled pilot studies	Outpatient mood disorders program of the Warneford Hospital & Oxford Health Foundation NHS Trust	120 days<br><br>Weekly ratings	Total:<br>19<br><br>Pilot 1:<br>14<br><br>Pilot 2:<br>5	37.2 (11.7)	31.6

and graphical reports							
ORBIT<br>Australia<br>Self-reported: Late stage bipolar disorder<br><br>Online strategies and exercises from acceptance and commitment therapy and mindfulness-based cognitive therapy	Murray et al [74]<br><br>2015	Uncontrolled pilot study	CREST.BD (international research & knowledge exchange network) website, Facebook, &Twitter pages	3 weeks<br><br>4 modules	26	46.6 (12.9)	25 (note: sex not obtained for 10 participants due to technical error)
Bipolar Education Program (BEP)<br>Australia<br>Clinician confirmed: Bipolar disorder<br>Online psychoeducation, mood charts, and support from 'informed supporters' via email	Nicholas et al [75]<br><br>2010	Qualitative analysis of RCT:<br><br>3 conditions: BEP;<br>BEP plus informed supporters (BEP + IS);<br>Attention control (AC)	Black Dog Institute Mood Disorders clinic, the Black Dog Institute website, community mental health organizations, general practitioners & psychiatrists, & the print media	8 weeks<br><br>8 modules	BEP:<br>16<br><br>BEP + IS:<br>9<br><br>AC:<br>14	NR	44
	Proudfoot et al [76]<br><br>2012	RCT:<br><br>3 conditions: BEP;<br>BEP + IS;<br>Attention control (AC)	Black Dog Institute Mood Disorders clinic, the Black Dog Institute website, community mental health	8 weeks<br><br>8 modules	BEP:<br>139<br><br>BEP + IS:<br>134<br><br>AC:<br>134	NR	BEP:<br>33.1<br><br>BEP + IS:<br>26.9<br><br>Attention control:

			organizations, general practitioners & psychiatrists, & the print media				30.6
The Netherlands<br>Clinician confirmed: Schizophrenia or axis 2 diagnosis with psychotic symptoms<br>In-person psychoeducation plus mobile text message goal prompts	Pijnenborg et al [77]<br><br>2010	Controlled trial:<br><br>Group 1:<br>A-B-A design (direct intervention - DI);<br>Group 2:<br>A-A-B-A design (waiting list - WL)	Referred by psychologists, psychiatrists, or nursing staff when impaired goal-directed behavior was observed	7 weeks	62<br><br>DI:<br>33<br><br>WL:<br>29	DI:<br>28.3 (8.0)<br><br>WL:<br>25.7 (7.5)	DI:<br>77<br><br>WL:<br>86
DBT Coach<br>US<br>Clinician confirmed: Borderline personality disorder with comorbid substance use disorder<br>Mobile dialectical behavior therapy	Rizvi, Dimeff, Skutch, Carroll, & Linehan[b] [31]<br>2011	Uncontrolled pilot study	3 outpatient DBT programs in the Pacific Northwest	10–14 days	22	33.86 (10.27)	18.2
SOAR<br>US<br>Clinician confirmed: Schizophrenia or schizoaffective disorder<br>In-person	Rotondi et al[b] [78,79]<br>2005, 2010	RCT:<br><br>2 conditions:<br>Telehealth website;<br>Usual care	Referred by staff at inpatient & outpatient psychiatric rehabilitation centers & psychiatric care units	N/A	Total:<br>31<br><br>Telehealth:<br>16<br><br>Usual care:<br>15	38 (11)	Telehealth:<br>38<br><br>Usual care:<br>27

psychoeducation followed by online psychoeducation, therapy groups, and questions via email							
	Rotondi, Eack, Hanusa, Spring, & Haas [80]<br>2015	Usability testing of SOAR compared with other websites	6 community mental health outpatient psychiatric rehabilitation centers	N/A (usability testing)	38	42.7 (6.62)	50
MyRecoveryPlan<br>US<br>Self-reported: Bipolar disorder<br>Online psychoeducation	Simon et al [81]<br>2010	Randomized controlled pilot study:<br><br>2 conditions: Program plus peer coaching; Program only	Email announcement to local Depression & Bipolar Support Alliance (DBSA) chapters, DBSA website, sponsored advertisement on Google, invitations from participants to peers, & cards in mental health clinics	Program available for 4–9 months (depending on sign-up time)	Total:<br>118<br><br>Coaching group:<br>64<br><br>Program only:<br>54	NR	28
Living with Bipolar (LWB)<br>UK<br>Confirmed by clinical questionnaire: Bipolar disorder<br>Online psychoeducation and CBT	Todd, Jones, & Lobban [82]<br>2012	Focus groups	Advertisement presented at self-help groups in the North West of England & Internet discussion forums	N/A (focus groups)	12	42 (8.85)	58.33
	Todd, Jones, Hart, & Lobban. [83]<br>2014	RCT:<br><br>2 conditions: LWB plus TAU; Waiting list	Voluntary sector organizations & online advertisements	6 months	Total:<br>122<br><br>LWB:<br>61	LWB:<br>42 (10.35)<br><br>WL:<br>45 (11.97)	LWB:<br>26<br><br>WL:<br>30

		control (WL)			WL:<br>61		
WEGWEIS<br><br>the Netherlands<br><br>Clinician confirmed: Schizophrenia or related psychotic disorder<br><br>Online routine outcome monitoring and personalized advice	van der Krieke, Emerencia, Aiello, & Sytema[b] [84]<br>2012	Pilot usability testing	4 mental health care organizations referred by clinicians & other study participants	N/A (usability testing)	15	42	NR
	van der Krieke et al[b] [85]<br>2013	RCT:<br><br>2 conditions: WEGWEIS; TAU	2 outpatient teams for psychosis: early intervention for psychosis team & a rehabilitation team	6 weeks	Total:<br>250<br><br>WEGWEIS:<br>124<br><br>TAU:<br>126	WEGWEIS:<br>37 (12.35)<br><br>TAU:<br>40 (12.7)	WEGWEIS:<br>67<br><br>TAU:<br>36
US<br>Clinician confirmed: Bipolar disorder<br>Mobile assessment & semi-individualized automatic feedback	Wenze, Armey, & Miller [86]<br>2014	Uncontrolled pilot study	Private psychiatric hospital: inpatient, partial hospital, and outpatient settings	2 weeks	14	40.86 (12.5)	28.57

[a] Study also included participants with different diagnoses; results for these participants are not reported in this review.

[b] Study included data from caregivers and/or clinicians; results from these participants are not reported in this review.
