# Supplementary material for: Acceptability of Interventions Delivered Online and Through Mobile Phones for People Who Experience Severe Mental Health Problems: A Systematic Review
Source: J Med Internet Res. 2016 May 31;18(5):e121. doi: 10.2196/jmir.5250 (PMC4908305; doi:10.2196/jmir.5250)
Supplement: Multimedia Appendix 3 [file jmir_v18i5e121_app3.pdf]

Table 3. Results and predictors relating to the hypothetical acceptability of online and mobile phone-delivered interventions for people with severe mental health problems.

Study reference(s)	Key acceptability outcomes	Demographic characteristics	Email vs text message delivery
Ben-Zeev et al (2013) [34,40], personal communication with D Ben-Zeev, May 2015	Bipolar disorder (n = 333):<br>137/333 interested in check-ins (41%);<br>129/333 interested in appointment and medication reminders (39%);<br>103/333 interested in psychoeducation and information (31%);<br>119/333 preferred text message delivery (36%);<br>271/333 preferred phone calls (81%);<br>70/333 preferred email delivery (21%).<br>Schizophrenia/schizoaffective disorder (n = 904):<br>344/904 interested in check-ins (38%);<br>398/904 interested in appointment and medication reminders (44%);<br>280/904 interested in psychoeducation and information (31%)	NR[a]	Higher interest in text message delivery (36%) than in email (21%).
Birnbaum et al (2015) [41], personal communication with A Rizvi, May 2015	Bipolar disorder (n = 11):<br>7/11 willing to have clinicians to approach via social media (64%);<br>8/11 liked idea of obtaining help via social media (73%).<br>Psychotic disorder (n = 40):<br>26/40 willing to have clinicians to approach via social media (65%);<br>31/40 liked idea of obtaining help via social media (78%).	NR	NA[b]
Bogart et al (2014) [42]	n = 85:<br>50/85 interested in text message medication reminders after discharge (59%)	Interest not significantly associated with age (*P*=.06), sex (*P*=.47), employment status (*P*=.93)	NA
Jain et al (2015) [43]	n = 201:<br>54/201 interested in medication and appointment reminders (27%);<br>29/201 interested in check-ins (14%);	NR	Text messages (8.5%) preferred over emails (1.5%)

	89/201 interested in information about services (44%);<br>146/201 preferred telephone calls (73%);<br>17/201 preferred text messages (8%);<br>3/201 preferred emails (1%)		
Lal et al (2015) [44]	Strongly agree/agree with the idea of using social media for information and/or support (n = 67):<br>57/67 YouTube (85%);<br>39/67 Facebook (58%);<br>27/67 Skype (40%);<br>26/67 Twitter (39%).<br>Strongly agree/agree with types of services delivered online or via mobile phones (n = 67):<br>62/67 appointment reminders via text (92.5%);<br>53/67 appointment reminders via email (79.1%);<br>49/67 appointment reminders via apps (73.1%);<br>45/67 medication reminders via text (67.1%);<br>38/67 medication reminders via email (56.7%);<br>42/67 medication reminders via apps (62.7%);<br>64/67 information about medications and associated side effects (95.5%);<br>62/67 information or support for education or employment, and tools to help decision making for treatment and recovery (92.5%);<br>61/67 information about psychosis and physical health (91%);<br>57/67 contacting team responsible for treatment (85.1%);<br>56/67 scheduling appointments; information about coping skills and events (83.6%);<br>44/67 online contact between clients (65.7%);<br>35/67 providing counselling or therapy services (52.2%)	NR	Reminders via text messages (92.5%/67.1%) preferred over reminders via email (79.1%/56.7%)
Miller et al (2015) [45]	Current text message users (n = 45):<br>20/45 would like to receive text messages from their doctors (45%);<br>26/45 would like to receive text messages with appointment reminders (58%);<br>18/45 would like to receive text messages with medication reminders	No associations between interest and age, sex, or race	Interest in text message services higher than interest in email services

	(40%);<br>20/45 would like to receive text messages asking about problems, symptoms, or side effects (44%)<br>Current email users (n = 30):<br>11/30 would like to receive emails from their doctors (37%);<br>13/30 would like to receive emails with appointment reminders (43%);<br>8/30 would like to receive emails with medication reminders (26%);<br>13/33 would like to receive emails asking about problems, symptoms, or side effects (43%)		
Sanghara et al (2010) [46]	n = 100:<br>76/100 willing to receive text messages from mental health service provider (76%)	NR	NA

[a]NR: not reported.

[b]NA: Not applicable.
