# Supplementary material for: Acceptability of Interventions Delivered Online and Through Mobile Phones for People Who Experience Severe Mental Health Problems: A Systematic Review
Source: J Med Internet Res. 2016 May 31;18(5):e121. doi: 10.2196/jmir.5250 (PMC4908305; doi:10.2196/jmir.5250)
Supplement: Multimedia Appendix 4 [file jmir_v18i5e121_app4.pdf]

Table 4. Results and predictors relating to the actual acceptability of online and mobile phone-delivered interventions for people with SMI measured by intervention use, module completion rates, and participant satisfaction.

Study reference(s)	Measures of acceptability		Demographic & clinical characteristics	Supported vs unsupported
	Intervention use & completion rates	Participant satisfaction		
Alvarez-Jimenez et al (2013)<br>[47]	12/20 - used HORYZONS for full 4 weeks (60%);<br>14/20 - used HORYZONS for ≥3 weeks (70%).<br><br>3/20 - completed all 7 modules (15%);<br>9/20 - completed ≥4 modules (45%);<br>12/20 - completed ≥3 modules (60%);<br>19/20 - completed ≥1 module (95%).	15/20 - had a positive experience (75%);<br>18/20 - would recommend to others (90%);<br>14/20 - felt it would be a useful long-term treatment option (70%).	Not reported (NR)	Not applicable (NA)
Gleeson et al (2013)<br><br>[48]	As above	20/20 - ‘agreed’ or ‘strongly agreed’ that HORYZONS was safe & confidential (100%);<br>18/20 - felt moderation had contributed to safety (90%).	NR	N/A
Kuosmanen et al (2009)<br><br>[50]	NR	All groups (n = 311):<br>3.09/4 - mean satisfaction score<br><br>IT education (n = 100):<br>3.16/5 - mean satisfaction score for meeting technical-scientific care needs;<br>3.01/4 - mean satisfaction score for meeting information care needs;<br>3.03/4 - mean satisfaction score for interaction/support care needs.	NR	N/A
Kuosmanen et al (2010)<br><br>[52]	NR	21/21 - agreed website contained relevant information (100%);<br>15/21 - agreed website provided new information (72%);<br>20/21 - agreed website was easy to use (95%)<br>16/18 - agreed website appearance was successful (90%)	NR	N/A
Pitkänen et al	IT education:	NR	NR	N/A

(2012)<br><br>[53]	87/100 - attended ≥3/5 sessions (87%).<br>Conventional education:<br>86/106 - attended ≥3/5 sessions (81%).			
Anttila et al (2012)<br><br>[54]	73/93 - attended all 6 sessions (79%).	NR	No relationship between session attendance &:<br>Age:<br>*P* = .576;<br>Sex:<br>*P* = .784;<br>Basic education:<br>*P* = .969;<br>Vocational education:<br>*P* = .976<br><br>No relationship between session attendance & Global Assessment of Functioning (GAF):<br>*P* = .093	N/A
Smith et al (2011)<br><br>[57]	13/24 (54.2%) - posted ≥1 message on discussion forum.<br><br>16/24 (66.6%) - completed ≥75% of the program (of total 47 subsections).	NR	NR	N/A
Barnes et al (2015)<br><br>[59]	Study group:<br>85/113 - completed 'majority' of sessions (75%).<br><br>Control group:<br>83/120 - completed 'majority' of sessions (69%).	NR	NR	N/A
Ben-Zeev et al (2013)<br><br>[40]	NR	10/12 - 'strongly agreed' or 'agreed' with ease of use (83%);<br>10/12 - found the system 'helpful' or 'somewhat helpful' (83%).	NR	NR
Ben-Zeev et al	Participants used FOCUS on 86.5% of the	30/32 - satisfied with ease of use (93.7%)	No relationship between	N/A

(2014)<br><br>[60]	study days (total study duration = 1 month):<br>Week 1: average use = 6.7/7 days<br>Week 2: average use = 5.9/7 days	29/32 - satisfied with focus (90.6%);<br>28/32 - would recommend to a friend (87.5%);<br>24/32 - would like to use FOCUS often (75%);	FOCUS use &:<br>baseline cognitive functioning, negative symptoms, & persecutory ideation<br>(all *P* > .05).	
Depp et al (2010)<br><br>[62]	78% - median percentage of completed surveys (2 week study duration; 4 daily prompts).	Participant ratings (n = 10):<br>9/10 – median satisfaction rating;<br>5/5 – median rating for ‘I would use this device again’;<br>5/5 – median rating for ‘this could be helpful to me in the future’.	NR	N/A
Depp et al (2015)<br><br>[63]	65% - compliance in PRISM condition (10 week study duration; 2 daily prompts).	Participant ratings (n = 41):<br>10/10 – median satisfaction rating;<br>5/5 – median rating for ‘I would use this device again’;<br>5/5 – median rating for ‘a device like this could help me’.	No relationship between compliance &:<br>Age:<br>*P* = .278;<br>Education:<br>*P* = .528<br><br>No relationship between compliance &:<br>Montgomery Asberg Depression Rating Scale (MADRS):<br>*P* = .717;<br>Young Manic Rating Scale (YMRS):<br>*P* = .451;<br>Illness Intrusiveness Scale (IIS):<br>*P* = .636	N/A
Depp et al (2010)<br><br>[62]	6/8 (75%) - showed ‘consistent engagement’ with texts (3 sets of 4 texts daily; 12 week study duration).	NR	NR	N/A
Granholm et al (2012)	Mean rate of valid assessment responses (3 sets of 4 texts daily; 12 week study duration; n = 42):	Participant ratings (n = 42):<br>3.15/4 – mean helpfulness rating	NR	N/A

[64]	Question 1 - medication adherence: mean = 86%;<br>Question 1 - socialization: mean = 83%;<br>Question 1 - auditory hallucinations: mean = 86%;<br>Question 2 - medication adherence: option 1 mean = 85%; option 2 mean = 85%;<br>Question 2 - socialization: option 1 mean = 78%; option 2 mean = 85%;<br>Question 3 - auditory hallucinations: option 1 mean = 85%; option 2 mean = 84%			
Forchuk et al (2015) [65], personal communication with L Warner, June 2015	NR	Mean satisfaction scores after 12 months:<br><br>Smartphone – psychotic disorder (n = 234):<br>5.8/7 – ease of use;<br>6.4/7 – helpfulness;<br>5.4/7 – simplicity.<br><br>Smartphone – personality disorder (n = 24):<br>4.9/7 – ease of use;<br>6.3/7 – helpfulness;<br>5.1/ - simplicity.<br><br>LSR – psychotic disorder (n = 234):<br>5/7 – ease of use;<br>5.5/7 – helpfulness;<br>5.1/7 – simplicity.<br><br>LSR – personality disorder (n = 24):<br>5.1/7 – ease of use;<br>5.6/7 – helpfulness;<br>3.9/7 – simplicity.	NR	N/A
Gottlieb et al (2013)<br><br>[66]	17/21 (81%) - completed ≥60% of the lessons (out of 10 total lessons).	Participant satisfaction ratings (n = 16):<br>82.4% - felt program was very good/helpful;<br>88.2% - felt right amount of information was covered;<br>88.2% - felt website was useful;<br>76.5% - would be very willing to recommend	NR	N/A

		to others;<br>52.9% found program very interesting.		
Holländare et al (2015)<br><br>[67]	NR	Participant satisfaction ratings (n = 4)<br><br>50% - fairly satisfied or very satisfied;<br>50% - neither satisfied nor dissatisfied.	NR	N/A
Kane et al (2013)<br><br>[68]	NR	Participant ratings (n = 27):<br><br>70% - found concept easy to understand;<br>78% - would like to receive mobile medication reminders;<br>89% - felt system could be useful.	NR	N/A
Lauder et al (2015)<br><br>[71]	n = 156:<br>48% - completed all 5 modules;<br>75.4% - completed ≥3 modules;<br>86.2% - completed ≥2 modules.	NR	NR	N/A
Lieberman et al (2011)<br><br>[72]	n = 64:<br>84/90 - mean number of days rated	NR	NR	N/A
Miklowitz et al (2012)<br><br>[73]	n = 19<br>Weekly text or emails sent for symptom ratings; duration 1–44 months:<br>81% - average percentage of texts or email prompts responded to.	NR	NR	N/A
Murray et al (2015)<br><br>[74]	NR	12/16 - would recommend to others (66.7%).	NR	N/A
Nicholas et al (2010)<br><br>[75]	All conditions:<br>160/358 - returned all 8 workbooks (44.7%);<br>263/358 - returned ≥4/8 workbooks (73.5%).<br><br>BEP + IS:<br>98/121 - returned ≥4/8 workbooks (81%);	NR	Males completed on average 0.98 fewer workbooks than females;<br>Participants >30 completed on average 1.04 more workbooks.	Adherence significantly higher in BEP + IS compared with BEP:<br>P = .01

	BEP:<br>80/120 - returned ≥4/8 workbooks. (66.7%).		Educational attainment did not predict workbook completion.<br><br>Levels of symptomology & baseline depression & anxiety scores did not predict workbook completion.	
Proudfoot et al (2012)<br><br>[76]	All conditions:<br>301/407 - returned ≥4/8 workbooks (74%);<br>158/407 - returned 8/8 workbooks (38.8%).<br><br>BEP + IS:<br>107/134 - returned ≥4/8 workbooks (79.9%);<br><br>BEP:<br>96/139 - returned ≥4/8 workbooks (69.1%);<br><br>Attentional control:<br>98/134 - returned ≥4/8 workbooks (73.1%).	NR	Relationship between number of workbooks returned &:<br>Age:<br>$P < .001$<br>Sex:<br>$P < .001$.<br><br>No relationship between number of workbooks returned & educational attainment.<br><br>Euthymia, depression scores, & anxiety scores did not predict number of workbooks returned.	Number of workbooks returned significantly higher in BEP + IS compared with BEP:<br>$P < .05$<br><br>Number of workbooks returned not significantly different between BEP + IS & attentional control or BEP & attentional control:<br>$P > .05$
Pijnenborg et al (2010)<br><br>[77]	NR	32/46 - gave positive evaluations (70%);<br>19/46 - felt text messages were effective (41%);<br>22/46 - were willing to continue with text messages after intervention finished (47%).	NR	N/A
Rizvi et al (2011)	Study duration: 10–14 days<br>85% - mean compliance with daily	22/22 - would use tool on own initiative (100%);	NR	N/A

[31]	assessments.	n = 176:<br>DBT coach was helpful 96.8% of the time;<br><br>Participant satisfaction ratings (n = 22):<br>4.05/5 – mean score for 'how likely is it that you would use this in your treatment?';<br>3.8/5 – mean score for overall helpfulness;<br>3.32/5 – mean score for enjoyment.		
Rotondi et al (2005, 2010)<br><br>[78,79]	16/16 (100%) - 'engaged' with treatment (contribution in forum on ≥13 visits & use of educational material on ≥4 visits).	11/16 - rated 'very much' or 'extremely' for ease of use (68.8%);<br>15/16- rated 'very much' or 'extremely' for value of website (93.8%).	Significant positive relationship between severity of positive symptoms & increased SOAR access:<br>*P* = .009;<br>increased SOAR usage:<br>*P* = .005	N/A
Rotondi et al (2015)<br><br>[80]	NR	Participant ratings (n = 38)<br>4.01/5 – mean rating for 'easy to use	NR	N/A
Simon et al (2010)<br><br>[81]	After 21 days:<br>Coaching group (n = 64):<br>24/64 - returned after sign-up (38%);<br>12/64 - started or updated any section of recovery plan (19%);<br>6/64 - used medication & side effects self-monitoring (10%);<br>5/64 - used goal progression self-monitoring (8%);<br>5/64 - used warning sign self-monitoring (8%);<br>10/64 - used discussion groups (16%);<br>7/64 - used peer-to-peer messages (11%)<br><br>Program-only group (n = 54):<br>5/54 - returned after sign-up (9%);<br>0/54 - started or updated any section of recovery plan (0%);<br>0/54 - used any self-monitoring tools & social	NR	NR	Coaching group showed significantly higher use of website after 21 days in all but 2 components.

	networking (0%).			
Todd et al (2014)<br><br>[83]	9/15 - average number of completed modules (60%).<br><br>n = 61:<br>29% - completed 1–5 modules;<br>31% - completed 6–14 modules;<br>34% - completed all 15 modules.	NR	NR	N/A
van der Krieke et al (2012)<br><br>[84]	NR	n = 15:<br>73.6/90 - mean satisfaction score;<br><br>13/15 - would recommend to others (86%);<br>12/15 - agreed website provided meaningful information (80%);<br>9/15 - agreed they would use website in the future (60%);	NR	N/A
van der Krieke et al (2013)<br><br>[85]	34/48 (71%) - used ‘full functionality’ of decision aid.	22/29 - felt well informed (76%);<br>22/29 - felt the advice helped them reflect (76%);<br>20/27 - would recommend to others (74%);<br>20/28 - easy to use (71%);<br>12/27 - said it helped them prepare to meet with clinicians (44%).	NR	N/A
Wenze et al (2014)<br><br>[86]	n = 14<br>25.64 /28 (91.57%) - average completion of sessions	Participant ratings (n = 14):<br>4.29/5 – average score for overall satisfaction;<br>4.25/5 – average score for helpfulness;<br>4.46/5 – average score for ease of use.	No significant relationship between completion rates &:<br>manic symptoms:<br>*P* = .77;<br>depressive scores:<br>*P* = .06 (association at 10% level)<br><br>No significant relationships between overall satisfaction or ease of use &:	

			manic symptoms:<br>*P* > .10;<br>depressive scores:<br>*P* > .10.<br><br>No significant relationship between perceived helpfulness &:<br>depressive scores:<br>*P* > .10;<br>manic symptoms:<br>*P* = .07 (association at 10% level).	
