# Supplementary material for: Acceptability of Interventions Delivered Online and Through Mobile Phones for People Who Experience Severe Mental Health Problems: A Systematic Review
Source: J Med Internet Res. 2016 May 31;18(5):e121. doi: 10.2196/jmir.5250 (PMC4908305; doi:10.2196/jmir.5250)
Supplement: Multimedia Appendix 5 [file jmir_v18i5e121_app5.pdf]

Table 5. Participant views and example quotes from studies that measured the acceptability of online and mobile phone-delivered interventions for people with SMI.

Paper reference	Participant views	Example participant quotes
Lederman et al (2014)<br><br>[49]	Participants felt HORYZONS:<br><br>1. gave them a sense of belonging, which made them feel safe & secure;<br><br>2. aided their understanding of psychosis;<br><br>3. helped them focus on strengths & fostered positive thoughts;<br><br>4. was visually engaging & appealing.	"…even though it was social networking, it was quite private—you didn't really have any information about yourself in the private profile apart from your name, and like say a photo…"<br><br>"… it gives you more of an understanding of things and I think if you understand what is happening to you, it makes it a little bit easier".<br><br>"… It's kind of like: hey, I was like that before, and hey look what I am now, and then I can help them. It makes you feel better that you can help other people too."<br><br>"...I like how you drag and drop the strengths. I've done card sorts before. So I am like hey, I've done this before".
Hätönen et al (2010)<br><br>[51]	Participants felt:<br><br>1. the procedure was systematic;<br><br>2. nurse-patient interaction was less mechanical when content was integrated into life situations with cooperative discussions;<br><br>3. the computer provided additional support for information delivery, but some were concerned about confidentiality;<br><br>4. interruptions would hinder program, while a peaceful environment would aid it;<br><br>5. information was to help with independent coping, while others felt the information was helpful but not useful for coping.<br><br>Participant suggestions:<br><br>1. individual participant situations should be considered when	"We had a schedule and in each session a specific topic was discussed".<br><br>"In what situation someone is, it is important. If someone is very tired or something, there should be a change to rest before those sessions".

	planning the patient education sessions;<br><br>2. interactive nature of IT should be used more. More visual and audio interactive components could have been included;<br><br>3. the environment for IT-based education should be peaceful;<br><br>4. computers should be on the ward to help facilitate and support information seeking.	
Anttila et al (2012)<br><br>[54]	Nurses reported that in sessions that they felt were successful:<br><br>1. patients were positive, motivated, interested, & enthusiastic;<br><br>2. patients' mental states were good & they understood the study;<br><br>3. patients' IT skills were good;<br><br>4. Oral patient feedback was positive.<br><br>In sessions that nurses felt were unsuccessful:<br><br>1. patients lacked interest & were more interested in different websites;<br><br>2. patients' mental states were poor or symptoms worsened during sessions & patients denied existence of their mental health problems;<br><br>3. patients' IT skills were poor or equipment did not function properly;<br><br>4. patients gave negative feedback – they would have liked to receive more information or could not find the answers to their questions.	Not reported (NR)
Laine et al (2015)<br><br>[55]	Phase 1:<br>Expectations:<br><br>1. content should include information, interactive tasks, a question & answer column, & a moderated discussion forum;<br><br>2. program and content should be reliable.	"Well those questions which specialists answer… if there is some question wondering and then he can ask that in that site and there is that kind of specialist who gives answers to those questions".<br><br>"I think that discussion forums should be utilized to hear what kind of symptoms and experiences others have with this illness".

	3. program should be easy to use, professional, and not contain too many pictures or colors.<br><br>Phase 2:<br>Proposals made by participants:<br><br>1. discussion forum;<br><br>2. information about money, anxiety, & manners;<br><br>3. smartphone app.	"I wouldn't ask just anybody in the Internet to be sure the information is reliable".<br><br>"That kind of user interface that it is easy to use and that you can find everything easily and things are not messed to everywhere".
Barnes et al (2011)<br><br>[56]	4 key themes:<br><br>1. concerns about individual differences, so modules shortened & available for longer;<br><br>2. stigma reduction & support;<br><br>3. issues with design;<br><br>4. relapse prevention.	"I think that would be ideal actually because then you could delve as deeply as you actually wanted to go".<br><br>"I think that what is reassuring though is the idea of a thermostat going wrong is that when you're depressed you think it's all your fault and you are personally responsible, you lack moral fibre, […] I think you've got to take away this horrendous drain you feel, that's what's very reassuring when you get help, that it's not all your fault".
Poole et al (2012)<br><br>[58]	Key themes:<br><br>1. content clarity & quality;<br><br>2. dislike of actors;<br><br>3. Difficulties with the life chart<br><br>4. lack of forum activity;<br><br>5. dislike of presentation of lithium;<br><br>6. alternatives to computer-based program;<br><br>7. lack of social opportunities in online psychoeducation, but group psychoeducation can be unappealing.	"… I enjoyed the clarity of the content and the way there was a lot of […] information available at many levels […] at every level of possible understanding, and it was very up to date as well".<br><br>"I didn't like the staged-ness […] you could tell they'd done it so many times they were probably on take 500 because someone had forgotten their lines, and it lost a little bit of its authenticity…".<br><br>"I can remember a timeline […] that did kerfuffle me a bit, remembering back all the bad stuff, wasn't good…"<br><br>"I think initially there was only 2 of us putting things back and forth and I think once we realized we were the only 2 we quickly retreated as well…"<br><br>"The one criticism I would have is that they were pushing lithium rather

	50% - would prefer group-based face-to-face psychoeducation;<br>50% - would prefer Beating Bipolar.	too much […] I though well maybe that's a little bit biased…so I thought maybe it was some sort of um pharmaceutical company that was involved with that […]".<br><br>"I suppose I just like more face-to-face stuff, […] I mean I'm 63, it's the younger generation that's much more accepting of this technology…"<br><br>"Personally I'd be more sort of geared towards learning with others and learning from others […] it's just because I don't ever talk about it in my day to day life with anyone so it's nice to be able to have people you can openly talk about it to".
de Leeuw et al (2012)<br><br>[61]	Before intervention:<br><br>1. participants expected it would help communication with caregivers & other patients;<br><br>2. some were concerned about privacy & reduced face-to-face contacts.<br><br>9-month follow-up:<br><br>Advantages of PCR:<br><br>1. accurate & reliable information about schizophrenia;<br><br>2. ability to view information about their treatment, medication, & relapse prevention.<br><br>Disadvantages of PCR:<br><br>1. privacy issues;<br><br>2. technical aspects needed improving;<br><br>3. too much information about illness & treatment;<br><br>4. needed a more personalized content.	NR
Depp et al	Interviews revealed several themes:	"At first I thought it wouldn't be beneficial, but it did help me 'catch'

(2010)<br><br>[62]	 1. increased emotional awareness & use of self-management behaviors;<br><br>2. participants requested summaries of their data for themselves & practitioners;<br><br>3. participants requested the ability to enter text-based entries & a broader selection of self-management strategies;<br><br>4. participants were concerned about what to say to others if asked what the purposed of the device was.	myself when I started to feel down".<br><br>"Helped make me think about what I am doing and whether I am using my strategies".
Latalova et al (2014)<br><br>[69]	Module feedback<br><br>Module 1 – participants happy to focus on family history; biological basis of bipolar disorder discussed with ambivalence. Some liked inclusion of famous people, while others felt inferior;<br><br>Module 2 – most participants identified with the included symptoms & division of symptoms into groups helped them understand bipolar disorder;<br><br>Module 3 – participants interested in genetic vulnerability & family history of psychiatric problems, & were able to recognize stressful events prior to development of disorder;<br><br>Module 4 – participants mostly interested in medication side effects & many could recognize at least one medication myth;<br><br>Module 5 – only half the participants said they were able to keep the changes in their life plan;<br><br>Module 6 – participants felt identifying warning signs and triggers was extremely beneficial;<br><br>Module 7 – participants found it difficult to recognize influence of thoughts on moods & practicing changing these thoughts;<br><br>Module 8 – participants found it difficult to recognize attitudes &	NR

	understand the impact that these attitudes can have on their lives;<br><br>Module 9 – participants enjoyed discussing relationships between their personality traits and stress, but found the adaptive attitudes component complicated;<br><br>Module 10 – participants liked this module, which focused on improving positive assertivity. However, they experienced problems with deciding where & when to use these skills & creating examples;<br><br>Module 11 – many participants disliked accepting criticism & criticizing others;<br><br>Module 12 – many participants felt it was important to have a person available for support if they experience a relapse.	
Lauder et al (2013)<br><br>[70]	Participant feedback:<br><br>1. mood monitor was useful;<br><br>2. satisfied with the flash object used for the key points;<br><br>3. liked having the information delivered sequentially.	NR
Murray et al (2015)<br><br>[74]	Participant feedback:<br><br>Participants were generally satisfied & positive about the program.<br><br>8 participants specified a preference for the inclusion of video media, while 2 preferred text;<br>8 participants felt the 3 week duration was not long enough;<br>15 reported no negative effects, but 1 was distressed by the 30 minute 'body scan'.	"As I am often online anyway, this venue was both convenient and relevant".<br><br>"I found the guided meditations quite helpful".<br><br>"When I was supposed to be applying things, I was still trying to learn things and complete homework…"<br><br>"Although mindfulness was helpful in identifying distress, it was not enough to help me cope with the emotional triggers that were released".
Nicholas et al (2010)<br><br>[75]	Key themes for nonadherence:<br><br>1. Unable to complete modules when experiencing acute symptoms;<br><br>2. Did not want to think about bipolar disorder;	"A very short while after doing the program I fell into another episode, a depressive episode, and pretty much stopped doing everything, the program included".<br><br>"I often go walking when having highs because I have to keep moving,

	3. Information was to basic;<br><br>4. Some stopped using it because they felt well or had gained what they wanted from it or used the information, but chose not to complete workbooks;<br><br>5. Time-related factors	so I didn't want to sit at a computer".<br><br>"I found it quite confronting, and reading he information made me feel uncomfortable, thinking that these issues related to me—I preferred the ostrich approach".<br><br>"The information in the modules was too general and too limited".<br><br>"I wanted something more about me specifically, as opposed to talking about general issues".<br><br>"I was so self-absorbed at the time that I was only interested in the information [rather than in returning workbooks]".<br><br>"I didn't have the time, and with everything else, it wasn't a priority".
Todd et al (2012)<br><br>[82]	Key themes:<br><br>1. Recognizing & managing mood swings;<br><br>2. There's too much information about symptom management online & not enough about living with bipolar disorder;<br><br>3. Participants did not want to stop mood swings altogether;<br><br>4. The Internet was the 'only format' for a self-management intervention;<br><br>5. The need for support from peers & professionals to provide motivation.	"…recognizing when I am going from one level of depression or mania to another more serious level where more intervention is needed on my own behalf or from outside help".<br><br>"… but there is so many (websites) out there they all resolve around the management of the illness… I'd like to see something that would deal with how to live your life as somebody who suffers from Bipolar".<br><br>"I value my mood swing, I rather like it… we don't want it absolutely flat. We don't want it absolutely perfect. We want an interesting life …, we want to maintain our relationships, quality of life and we want to be darn useful to someone".<br><br>"It's the only format… the Internet format allows people wot freely access it rather than having to wait for interventions through the NHS…"<br><br>"Something you can interact with is much better. Reading a book for a start off, if you are manic or depressive ready is really difficult, really difficult…"<br><br>It's really hard for me to sustain being involved in something for a long time depending on where my mood changes… there has been times when I really need to do that again and I know it works but for some

		reason I just couldn't motivate myself to do it…"
van der Krieke et al (2012)<br><br>[84]	1. some of the buttons were hard to find;<br><br>2. some participants wanted the website to be more attractive, but others were happy with the layout;<br><br>3. some suggested further information about symptoms & medication.	NR
van der Krieke et al (2013)<br><br>[85]	Participants experienced differences in how the website was presented to them by case managers.<br><br>Most participants could not remember a treatment plan being created or routine outcome monitoring results being discussed.	NR
